# How Does Salinity Shape Bacterial and Fungal Microbiomes of *Alnus glutinosa* Roots?

**DOI:** 10.3389/fmicb.2018.00651

**Published:** 2018-04-18

**Authors:** Dominika Thiem, Marcin Gołębiewski, Piotr Hulisz, Agnieszka Piernik, Katarzyna Hrynkiewicz

**Affiliations:** ^1^Department of Microbiology, Faculty of Biology and Environmental Protection, Nicolaus Copernicus University in Toruń, Toruń, Poland; ^2^Centre for Modern Interdisciplinary Technologies, Nicolaus Copernicus University in Toruń, Toruń, Poland; ^3^Chair of Plant Physiology and Biotechnology, Faculty of Biology and Environmental Protection, Nicolaus Copernicus University in Toruń, Toruń, Poland; ^4^Department of Soil Science and Landscape Management, Faculty of Earth Sciences, Nicolaus Copernicus University in Toruń, Toruń, Poland; ^5^Chair of Geobotany and Landscape Planning, Faculty of Biology and Environmental Protection, Nicolaus Copernicus University in Toruń, Toruń, Poland

**Keywords:** salinity, ectomycorrhiza, arbuscular mycorrhiza, *Frankia* sp., metagenomics, Illumina MiSeq

## Abstract

Black alder (*Alnus glutinosa* Gaertn.) belongs to dual mycorrhizal trees, forming ectomycorrhizal (EM) and arbuscular (AM) root structures, as well as represents actinorrhizal plants that associate with nitrogen-fixing actinomycete *Frankia* sp. We hypothesized that the unique ternary structure of symbionts can influence community structure of other plant-associated microorganisms (bacterial and fungal endophytes), particularly under seasonally changing salinity in *A. glutinosa* roots. In our study we analyzed black alder root bacterial and fungal microbiome present at two forest test sites (saline and non-saline) in two different seasons (spring and fall). The dominant type of root microsymbionts of alder were ectomycorrhizal fungi, whose distribution depended on site (salinity): *Tomentella*, *Lactarius*, and *Phialocephala* were more abundant at the saline site. *Mortierella* and *Naucoria* (representatives of saprotrophs or endophytes) displayed the opposite tendency. Arbuscular mycorrhizal fungi belonged to Glomeromycota (orders Paraglomales and Glomales), however, they represented less than 1% of all identified fungi. Bacterial community structure depended on test site but not on season. Sequences affiliated with *Rhodanobacter*, *Granulicella*, and *Sphingomonas* dominated at the saline site, while *Bradyrhizobium* and *Rhizobium* were more abundant at the non-saline site. Moreover, genus *Frankia* was observed only at the saline site. In conclusion, bacterial and fungal community structure of alder root microsymbionts and endophytes depends on five soil chemical parameters: salinity, phosphorus, pH, saturation percentage (SP) as well as total organic carbon (TOC), and seasonality does not appear to be an important factor shaping microbial communities. Ectomycorrhizal fungi are the most abundant symbionts of mature alders growing in saline soils. However, specific distribution of nitrogen-fixing *Frankia* (forming root nodules) and association of arbuscular fungi at early stages of plant development should be taken into account in further studies.

## Introduction

The global scarcity of water resources, environmental pollution and increased salinization of soil and water are the most pressing problems of the 21st century. Salinity affects more than 7% of global land surface and 70% of all irrigated agricultural soils worldwide (Bencherif et al., 2015). Current predictions indicate that salinity is expected to be responsible for 30% land loss within the next 25 years, and up to 50% within the next 35 years (Chandrasekaran et al., 2014). Afforestation may contribute to solving the problem of soil reclamation, as it is a sustainable land use system that serves as an alternative to agriculture. In general, trees are more tolerant to salt stress than herbaceous crops (Chen et al., 2014). Among them, representatives of Betulaceae family are the most widely planted around the world for the rehabilitation of salinity affected lands (Diagne et al., 2013).

*Alnus glutinosa* Gaertn., commonly known as black alder, belongs to this plant family and is an actinorrhizal plant that forms symbiosis with nitrogen-fixing actinomycete *Frankia* sp. (McEwan et al., 2017; Roy et al., 2017). Additionally, *A. glutinosa* can be colonized by both ectomycorrhizal (EM) and arbuscular (AM) fungi at the same time (Pritsch et al., 1997). *Alnus* sp. has high potential in forestry, land reclamation and biomass production (Roy et al., 2007). Alder trees belong to pioneer tree species and can grow in poor or disturbed soils and are well adapted to abiotic stresses, e.g., drought, salinity, and flooding (Diagne et al., 2013).

The ability of alders to form multifactorial symbiotic association can be a key factor improving their tolerance to saline stress conditions. The symbiotic relationship with *Frankia* sp. increases the soil fertility and enhances the performance of tree during their plantation under unfavorable conditions (Diagne et al., 2013). *Alnus* plants inoculated with *Frankia* sp. in general display improved plant growth, total biomass, nitrogen supply, and chlorophyll content in leaves tissues under saline conditions (Oliveira et al., 2005; Ngom et al., 2016). However, the selection of tolerant *Frankia* sp. strains is needed because this symbiosis is facultative for the actinobacteria (Põlme et al., 2013). *Frankia* strains vary in their sensitivity and response to salinity (Ngom et al., 2016) and the number of root nodules, vesicle production and nitrogenase activity is affected under saline stress conditions (Oshone et al., 2013).

Mycorrhizal fungi – ectomycorrhizal and arbuscular – play an important role in protection of plants against abiotic stress in the environment (Schützendübel and Polle, 2002; Meharg, 2003; Ruotsalainen et al., 2009). Mycorrhizal fungi prevent Na^+^ and Cl^-^ translocation to shoot and leaf tissues and enhance nutrients uptake, e.g., phosphorus (P) and nitrogen (N), in moderately salt-tolerant plants, e.g., black alder (Tang et al., 2009; Evelin et al., 2012), which indirectly increases plant growth and subsequent diminution of toxic ion effects (Tang et al., 2009). Mycorrhiza indirectly increase salinity tolerance by other mechanisms, e.g., by synthesis of phytohormones, amelioration of rhizosphere and bulk soil conditions (Asghari et al., 2005), enhancement of photosynthetic activity, water uptake (Hajiboland et al., 2010), accumulation of compatible solutes (Evelin and Kapoor, 2013), and production of higher levels of antioxidant enzymes (Manchanda and Garg, 2011). However, saline stress can affect mycorrhizal association (Hrynkiewicz et al., 2015) by decreasing fungal colonization capacity, the growth of fungal hyphae and germination of fungal spores in soil (Hameed et al., 2014). A broad taxonomic range of EM fungi can grow at ∼170 mM NaCl, although at a lower rate than in the absence of salt (Chen et al., 2001). This fact can suggest high salt resistance of many EM fungi (e.g., Dixon et al., 1993). Many reports describe successful application of salt-tolerant mycorrhizal fungi in plants protection against saline stress (Langenfeld-Heyser et al., 2007; Estrada et al., 2013; Talaat and Shawky, 2014; Sarwat et al., 2016). High concentrations of soluble salts have negative effect on microorganisms, e.g., decrease microbial activity and biomass and affect microbial community structure (Yan et al., 2015). However, this environmental factor can also predominantly stimulate occurrence of some salt tolerant bacterial and fungal strains, and the knowledge of changes in microbial community under salt stress conditions can be useful in development of new technologies used in forestry.

The aim of our study was to assess the effect of the unique ternary structure of alder symbionts on community of other root-associated microorganisms (bacterial and fungal endophytes), particularly under seasonally changing salinity. Specifically, we hypothesize that: (i) one type of symbiosis will dominate, (ii) salinity and seasonality will affect diversity and species richness of plant-associated bacteria and fungi, and (iii) salinity can preferentially promote occurrence of certain groups of symbionts or halotolerant bacterial and/or fungal taxa. Broadening of the knowledge on alder’s endophytes can open new horizons in plant-microbial interactions occurring in forests.

## Materials and Methods

### Site Description and Sampling

The study was carried out in mid-northern Poland at two test sites: a non-saline one in Pszczółczyn (53° 00′22.9″ N, 17° 54′57.2″ E; NS) and a saline one in Słonawy (53° 01′26.6″ N, 17° 37′47.3″ E; S) (Thiem et al., 2017). Salinity of soils in this area results from the impact of saline springs contacting with the Zechstein (Permian) salt deposits (Dadlez and Jaroszewski, 1994). The investigated saline site is located in the close vicinity of salt marshes (Solniska Szubińskie) belonging to the European Ecological Network Nature 2000. Analyzed sites belong to the State Forests, outside of any protected areas. The control site Pszczółczyn (NS) is located 18 km away from the saline test site (S). Both sites are located in Odra drainage basin, elevation is 100 m over the sea level and the sites are flat (<1° slope). They are characterized by similar climatic, hydrological and pedological conditions and harbor tree stands of similar age [about 20 years old, planted in 1995 (S) and 1996 (NS), respectively]. Climate is temperate, mean annual temperature is 8.4°C, and precipitation 520 mm. Groundwater table usually lies at lower than 1 m depth, the sites are located ∼1.5 km from the nearest river, and as such are not flooded by river waters. Soils are mineral-mucky developed on sands. Groundcover at the NS site is typical for riparian forest and consists mainly of *Urtica dioica*, *Stachys sylvatica*, and *Ranunculus lanuginosus*, but also *Rubus occidentalis* could be found. At the S site the groundcover was even less diverse consisting almost exclusively of *U. dioica*.

Roots and soil samples (20 cm × 20 cm, 20 cm depth, litter layer was first removed) were collected from two test sites (NS and S) in two seasons of 2015 (spring – April and fall – September). In each test site three plots (10 m × 10 m) were selected, and within them three randomly selected trees were analyzed (9 trees per site). In total, 36 samples were analyzed (9 × 4 variants of experiment: NS-spring, NS-fall, S-spring, S-fall) (Supplementary Figure 1).

### Soil Description and Analysis

The air-dried soil samples were passed through a 2 mm mesh and analyzed using the following methods: soil moisture content – gravimetrically, total organic carbon (TOC) and total nitrogen (TN) content using a CNS Vario MAX analyzer, pH (in H_2_O and 1 M KCl) by potentiometric method, and phosphorus in 1% citric acid solution (P_ca_) by colorimetric method. The saturation paste extracts were prepared to evaluate the soil salinity level. The electrical conductivity (EC_e_) was measured conductometrically and saturation percentage (SP) – gravimetrically (van Reeuwijk, 2006). Soil moisture content (M) was determined by drying to constant weight at 105°C.

### Metagenomic Analysis

#### Roots Cleaning and Preparation

First, soil was gently separated from roots to obtain rhizosphere soil for analysis of physico-chemical properties. Next, the pre-cleaned roots were thoroughly washed with sterile distilled water. Residual soil was separated from the roots with a sterile dissecting needle under magnifying glass and the roots were washed again with sterile distilled water. All steps were performed under sterile conditions. Ca. 500 mg samples were randomly collected from pools of cleaned roots and lyophilized.

#### Isolation of Metagenomic DNA

Total DNA was extracted from 50 mg of lyophilized black alder roots with the use of Plant & Fungi DNA Purification Kit (EURx, Poland) according to the manufacturer’s protocol with the number of washing steps increased to four. Three technical replicates (independent DNA isolations) were prepared for each sample. Tubes with sterile glass beads (Mo Bio Laboratories) were used for plant material homogenization. The amount of isolated DNA was quantified fluorometrically (Qubit 2.0) and the quality was assessed spectrophotometrically (NanoDrop 2000) and the preparations were diluted to 1 ng/μl.

#### PCR Amplification of 16S rRNA Gene as Well as ITS Fragments and Sequencing

Bacterial 16S rRNA and fungal ITS amplicon libraries were generated in two-step PCR, first with the specific primers bearing M13/M13R overhangs (Gołębiewski et al., 2014) (bacteria: u357f and u786r; fungi: uITS1 and uITS2) then with M13 and M13R primers with P5/P7 adapters and barcodes (different MID sequences for each sample) (**Table 1**). Mock community sample with known composition of bacterial species (Human Microbiome Project Mock Community A, BEI Resources) was also processed. Negative control (without DNA) and positive control (*Escherichia coli* K12 DH10B [NC_00930.1] and *Paxillus involutus* [GQ389624.1] purified DNA) were included in each PCR round.

**Table 1:** Primer sequences.

Name1	Sequence 5′ → 3′2	Paired with	Target	Reference
u357f	GTTTTCCCAGTCACGACCCTACGGGAGGCAGCAG	B786rU	Bacterial 16S	Neefs et al., 1993
u786r	CAGGAAACAGCTATGACACCAGGGTATCTAAWCC	B357fU		Deja-Sikora, 2012
uITS1	**GTTTTCCCAGTCACGA**CTCCGTAGGTGAACCTGCGG	uITS2	Fungal ITS	This work
uITS2	**CAGGAAACAGCTATGAC**TTYGCTGYGTTCTTCATCG	uITS1		This work
M13-x-A3,4	CCATCTCATCCCTGCGTGTCTCCGAC*TCAG*XGTTTTCCCAGTCACGAC	M13R-B	M13-tagged amplicons	This work
M13-x-A3,4	CCATCTCATCCCTGCGTGTCTCCGAC*TCAG*XGTTTTCCCAGTCACGAC	M13R-B		This work


**1 – u denotes M13/M13R tagged sequences; 2 – M13 and M13R sequences are given in boldface font; 3 – key sequence in italics; 4 – X denotes barcode (MID sequence)*.*

The first PCR reaction mix consisted of: 1 ng of DNA, 5 pmol of each primer, 4 nmol of each dNTP, 0.4 U of Phusion polymerase (Thermo Scientific), 100 μg of BSA and 1× concentrated buffer with 1.5 mM MgCl_2_ in 20 μl. The cycling conditions were as follows: 98°C – 30 s; 30 cycles of: 98°C – 10 s, either 55°C (bacteria) or 53°C (fungi) – 15 s, 72°C for 20 s; then 5 min at 72°C. The PCR products were checked on 1.5% agarose gels in TBE and then they were purified using DNA Clean-Up Purification Kit (EURx) according to the manufacturer’s protocol. Next, PCR products were quantified on Qubit 2.0 and diluted to 1 ng/μl.

The second PCR round was performed using Taq PCR Master Mix Kit (Qiagen) according to the manufacturer’s protocol. The cycling conditions were as follows: 95°C – 5 min, 14 cycles of: 95°C – 30 s, 54°C –15 s, 72°C – 30 s; and finally 72°C for 1 min. The products were checked again on 1.5% agarose gel, quantified with Qubit 2.0 (Thermo Scientific) and pooled in equimolar amounts.

Libraries were purified twice with Agencourt AMPure XP (Beckman Coulter) according to the manufacturer’s protocol. The quality of the pooled libraries was assessed on a Bioanalyzer chip (Agilent) and they were quantified with KAPA Library Quantification Kit for Illumina Platform using LightCycler 480 (Roche) according to the manufacturers’ protocols. The final pool was diluted to 4 nM, denaturated, mixed with 5% of PhiX control library and sequenced with the use of 2 × 300 cycles kit v.3 on a MiSeq machine (Illumina). Sequencing was performed using HPLC-purified versions of forward and reverse primers as well as reverse-complement of the reverse primer (**Table 1**).

#### Bioinformatic and Statistical Analyzes

The resulting read pairs were quality filtered with Sickle (Joshi and Fass, 2011), merged with Pandaseq (Masella et al., 2012) and denoised with BayesHammer (Nikolenko et al., 2013). The sequences were classified with naive Bayesian classifier (Wang et al., 2003) with SILVA Seed v.123 database then bacterial and non-bacterial ones were separated.

In case of the bacterial 16S rRNA sequences, the processing was performed essentially as described in Gołębiewski et al. (2014). In brief: the sequences set was dereplicated, aligned to a template alignment (SILVA v. 123) and screened for the sequences covering the desired region of the alignment. Gap-only and terminal gap-containing columns were filtered out of the alignment, the set was pre-clustered to reduce the error rate and putative chimeras were identified with UCHIME (Edgar et al., 2011). The fungal sequences were processed with ITSx (Bengtsson-Palme et al., 2013), and all fungal ITS1 sequences were used in the downstream analyzes. The reads were dereplicated and OTUs were constructed using vsearch (Rognes et al., 2016) at 0.03 dissimilarity level, then singletons as well as doubletons (OTUs consisting of one or two sequences only) were removed. The sequences were classified with naive Bayesian classifier (minimum 80% bootstrap support was required; Wang et al., 2003) using SILVA v.123 (bacteria) and ITS1 extracted from UNITE v.7 (fungi), and the non-bacterial and non-fungal sequences were removed from the respective sets. The final data were subsampled to 500 (bacteria) and 300 (fungi) sequences per sample twenty times, sequences names were mangled to reflect the iteration, the sets were pooled, dereplicated, and OTUs were constructed as described earlier. OTU tables were then averaged over the twenty subsamples and the entries were rounded to the nearest integer with a Perl script to yield the final tables. Bray–Curtis distance matrices based on Wisconsin double-standardized OTU tables were calculated with vegdist in R. Non-metric multidimensional scaling (NMDS) and canonical correspondence analysis (CCA) analyzes were performed within R with vegan’s metaMDS and cca functions. In case of CCA, forward selection procedure implemented in ordistep was used for model building. Significance of differences between sample clusters was assessed with ANOSIM and PERMANOVA in vegan’s anosim and adonis functions, respectively. *p*-value < 0.05 was considered significant. Variance partitioning was performed with the varpart function.

Differences between soil parameters were analyzed by the non-parametric Kruskal–Wallis test and the Dunn test for *post hoc* comparison (Statistica ver. 7.1, StatSoft et al., 2006). Significance of differences in means (number of observed OTUs, Shannon’s H’, Shannon’s E, taxa and functional groups distribution) was assessed with ANOVA with *post hoc* Tukey’s HSD analysis, unless assumptions of normality of data and/or homogeneity of variance were violated, in which case Robust ANOVA implemented in raov of the Rfit package was used to check for general *p*-value. All figures were plotted with standard R graphic functions.

Bacterial sequences were classified to functional groups (nitrogen-fixing, nitrifying, denitrifying, halotolerant/halophilic) based on a BLAST analysis. Matches were considered significant when the alignment spanned over 99% of query length and identity was over 99% (details in Supplementary Material).

Fungal sequences classified down to the species level were assigned to functional groups [saprophytic (S), parasitic/pathogenic (P), endophytic (E), mycorrhizal (M)] based on information contained in NCBI’s databases including source of isolation, host and data contained in references pertaining to a given sequence (details in Supplementary Material).

Possible metagenomes were imputed based on 16S rRNA fragments data with PICRUSt v.1.1.3 (Langille et al., 2013). Briefly, sequences were reclassified using Greengenes v.13_8_99 (DeSantis et al., 2006), then a BIOM file was produced with MOTHUR and made compatible with PICRUSt using BIOM tools^1^. Then, the BIOM file was normalized with normalize_by_copy_number.py and metagenomes were predicted as well as NSTI scores calculated with predict_metagenomes.py, using KEGG Orthology^2^ as the base for predictions. Finally, an.spf file was prepared with biom_to_stamp.py. Predicted metagenomes were analyzed at the KEGG Orthologs (KO) level with STAMP v.2.1.3 (Parks et al., 2014), using the following parameters: remove unclassified reads, analysis of two groups, two sided Welch’s test with Benjamini-Hochberg FDR correction. *Q*-value threshold was set to 0.01 and minimum ratio of proportions of five was used as a filter. PCA plot as well as barplot for the most significantly different categories were prepared.

## Results

### Soil Physico-Chemical Parameters Differ Between Test Sites and Seasons

Physico-chemical soil parameters of samples are presented in **Table 2**. The EC_e_ values at the S site ranged between 2.4 and 5 dS⋅m^-1^ in spring, and they were significantly lower (∼1 dS⋅m^-1^) in fall. At the NS site the values ranged between 0.55 to 1.13 dS⋅m^-1^. We supposed that the low EC_e_ values at the saline site in fall were due to heavy rainfalls occurring right before sampling, which caused increase of soil water content and decreased salinity. It was confirmed by the higher level of soil moisture (M) at the S site, close to the values of SP. Similar phenomenon was observed at the NS site (**Table 2**). The soil samples from the S site were more acidic (pH-H_2_O 6.1 and 5.6 in spring and fall, respectively) than the NS ones (pH-H_2_O 6.7 and 6.5 in spring and fall, respectively). Soils at both sites were mineral, but the nutrients content (TOC, TN, and P_ca_) was consistently higher at the NS site (**Table 2**).

**Table 2:** Physicochemical and chemical parameters of the studied soils (mean ± standard deviation) and Kruskal–Wallis test with the Dunn *post hoc* comparisons for analyzed variants (site: NS, non-saline; S, saline; season – fall, spring).

	NS		S		
			
Season	Spring	Fall	Spring	Fall	*p*
EC_e_ (dS⋅m^-1^)	0.90 ± 0.23^a^	0.80 ± 0.25^a^	3.70 ± 1.29^b^	1.10 ± 0.34^a^	0.0001
pH-H_2_O	6.5 ± 0.5^ab^	6.7 ± 0.3^b^	6.1 ± 0.7^ab^	5.6 ± 1.0^a^	0.0196
pH-KCl	6.1 ± 0.6^bc^	6.3 ± 0.4^c^	5.0 ± 0.8^ab^	4.3 ± 0.9^a^	0.0001
TOC (%)	8.18 ± 2.34^b^	9.90 ± 4.81^b^	3.74 ± 1.36^a^	5.31 ± 2.05^ab^	0.0005
TN (%)	0.72 ± 0.18^b^	0.84 ± 0.38^b^	0.36 ± 0.12^a^	0.49 ± 0.17^ab^	0.0004
P_ca_ (mg⋅kg^-1^)	90.8 ± 30.2^b^	83.6 ± 33.9^b^	36.7 ± 4.60^a^	34.7 ± 8.13^a^	0.0000
M (%)	37.4 ± 13.4^ab^	73.8 ± 15.4^c^	27.6 ± 7.68^a^	64.7 ± 22.6^bc^	0.0000
SP (%)	79.2 ± 20.3^a^	76.1 ± 22.5^a^	64.1 ± 12.8^a^	83.3 ± 26.7^a^	0.3318


**Variants labeled with the same letters are not significantly different (p ≤ 0.05). EC_*e*_, electrical conductivity of the saturated extract; pH-H_*2*_O – pH in water; pH-KCl, pH in 1M KCl; TOC, total organic carbon; TN, total nitrogen; P_*ca*_, phosphorus in 1% citric acid solution, M, soil moisture, SP, saturation percentage*.*

### Sequencing Statistics

3 158 772 read pairs were obtained, of which 2 317 906 met the quality criteria and were merged. Of these, 546 961 sequences were classified as bacterial, and the rest (1 770 945) was regarded to be ITS sequences. 319 355 putative chimeras and 43 184 doubletons and singletons were removed from the bacterial dataset, leaving 184 422 sequences in the analysis (1–29 056 per sample).

ITSx correctly identified fungal ITS1 region in 863 931 putative ITS sequences. After classification with naive Bayesian classifier using the UNITE database, 588 198 non-fungal sequences were culled. They originated mainly from the host (*Alnus glutinosa*), demonstrating that the primers amplify not only fungal sequences. Finally, after removal of 11 264 singletons and doubletons 264 499 sequences were included in downstream analyses (2–18 341 per sample).

The reads, bacterial and fungal separately, were deposited in NCBI’s SRA under accession no. SRP119174.

### Microbial Community – Species Richness and Diversity Indices

**FIGURE 1:**
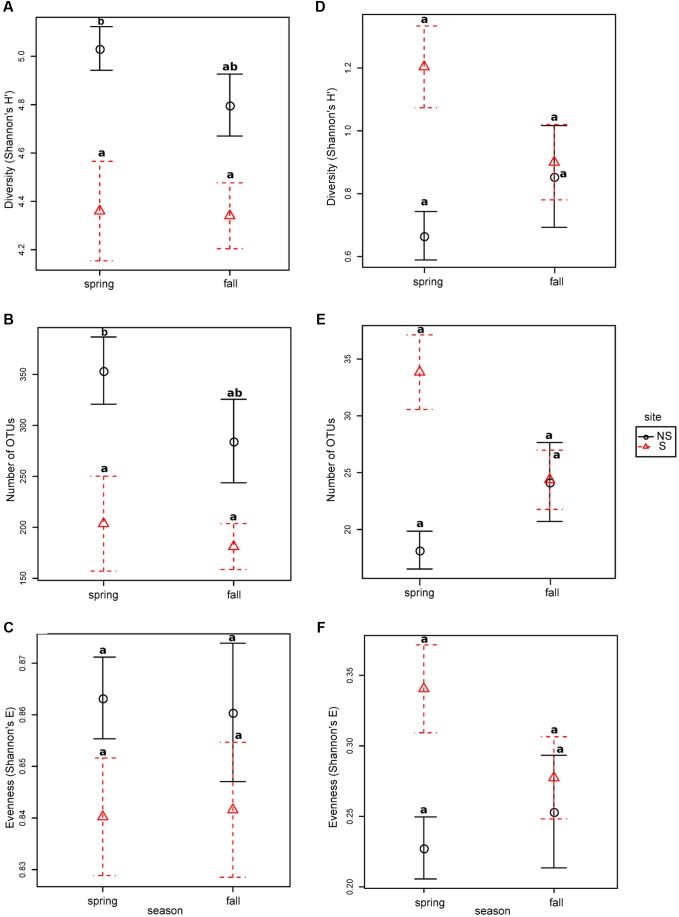
Species richness, diversity and evenness in different seasons and sites for OTUs constructed at 0.03 dissimilarity for bacterial and fungal sequences: panels **(A,D)** Shannon’s H’, **(B,E)** – observed number of OTUs, **(C,F)** – Shannon’s E. Robust ANOVA test with the Tukey’s *post hoc* analysis were used to assess significance of differences between sites and seasons. Variants labeled with the same letters are not significantly different (*p* ≤ 0.05).

Bacterial diversity, species richness as well as evenness for OTUs constructed at 0.03 dissimilarity threshold were lower at the S site both in spring and fall (**Figures 1A–C**). The opposite tendency was observed for fungal community, however, in fall the values were comparable at both sites, and they decreased at the S site and increased at the NS site (**Figures 1D–F**). Parameters measured for the bacterial community were always higher than for the fungal one: diversity ∼fourfold (bacteria: 4.3–5.0, fungi: 0.6–1.2), species richness ∼100 times (bacteria: 180–350, fungi: 18–34), and evenness ∼threefold (bacteria: 0.84–0.86, fungi: 0.21–0.34) (**Figure 1**).

### Bacterial and Fungal Community Structure

**FIGURE 2:**
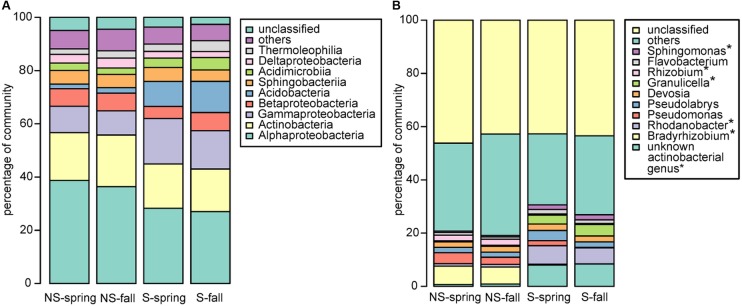
Bacterial community structure at the **(A)** class and **(B)** genus level. ^∗^significant differences in the distribution.

Bacterial community was dominated by bacteria belonging to proteobacterial classes Alphaproteobacteria (NS: 36.4–38.7%, S: 27.0–28.3%) and Gammaproteobacteria (NS: 9.1–9.9%, S: 14.4–17.1%), Actinobacteria (NS: 18.0–19.4%, S: 16.0–16.6%) and Acidobacteria (NS:1.7–2.1%, S: 9.4–11.7%) (**Figure 2A**), however, the observed differences between the test sites were not significant. To the contrary, significant differences were observed at the genus level, where the most abundant genera differed between the NS and S sites: *Bradyrhizobium* and *Rhizobium* were more frequent at the NS site, while an unknown actinobacterial genus, *Rhodanobacter*, *Granulicella*, and *Sphingomonas* were more abundant at the S site (**Figure 2B**). At the level of order, sites differed significantly in abundance of Frankiales, comprising bacteria of genus *Frankia*, and greater abundance was observed at the S site (Supplementary Figure 2B). No significant differences between seasons were observed for all taxonomic levels.

**FIGURE 3:**
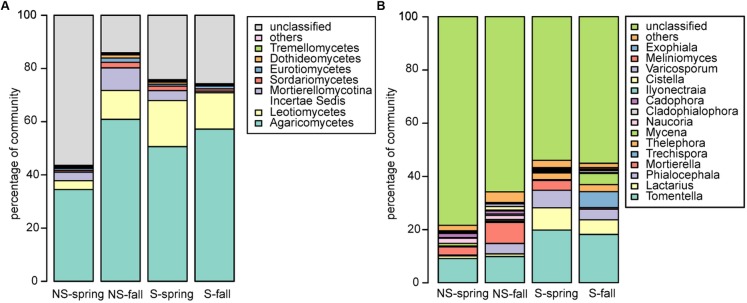
Fungal community structure at the **(A)** class and **(B)** genus level.

Fungal community was dominated by Agaricomycetes (NS: 34.5–60.9%, S: 50.6–57.2%), Leotiomycetes (NS: 3.3–10.9, S: 13.7–17.4%), Mortierellomycotina Incertae Sedis (NS: 3.2–8.5%, S: 0.6–3.7%), and Sordariomycetes (NS: 06–2.1%, S: 0.9–1.7%) (**Figure 3A**). Similarly to bacteria, differences between sites and seasons at the class level were not significant, however, in general, greater abundance of Agaricomycetes was observed in fall, and Leotiomycetes were more frequent at the S site, and at the NS site in fall, while Mortierellomycotina Incertae Sedis and Sordariomycetes were most abundant at the NS site in fall, and at the S site in spring. At the genus level, *Tomentella*, *Lactarius*, *Phialocephala*, and *Mortierella* were the most frequent taxa, however, their share depended on site. *Tomentella*, *Lactarius*, and *Phialocephala* were more abundant at the S site, while *Mortierella* and *Naucoria* displayed the opposite tendency (**Figure 3B**). *Tomentella*, *Lactarius*, *Thelephora*, and *Naucoria* were the most frequently identified ectomycorrhizal fungi in all variants of the experiment (**Figure 3B**). Smaller number of sequences was noted for other EM fungal genera, such as *Cortinarius* or *Amanita* that were confounded within ‘other fungi’ group due to the low number of reads. Arbuscular mycorrhizal fungi were found in all samples and represented less than 1% of all identified fungi in each of them. They belonged to Glomeromycota (orders Paraglomales and Glomales, data not shown).

### Microbial Communities Ordinations

**FIGURE 4:**
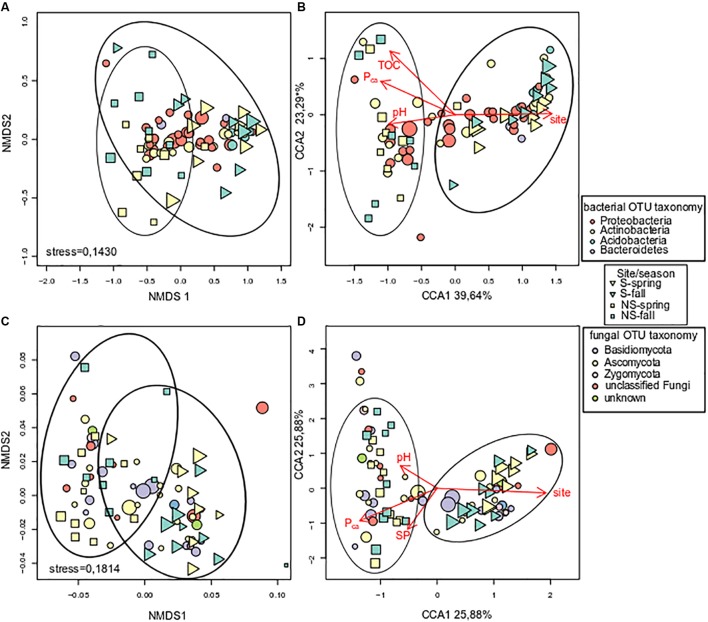
Analysis of log-transformed and Wisconsin double-standardized Bray–Curtis dissimilarity matrix for bacterial and fungal communities, respectively: **(A)** stress = 0.1430 and **(C)** stress = 0.1814 – NMDS (non-metric multidimensional scaling analysis), **(B,D)** – CCA (canonical correspondence analysis). Circles represent OTUs, their fill color denotes consensus taxonomy at the phylum level. Fifty most abundant OTUs were plotted in each case. Squares and triangles represent sites, their fill color means season. Area of figures is proportional to square root of EC_e_ (electrical conductivity) in soil samples.

Unconstrained ordination of bacterial community matrix (Bray–Curtis distance-based NMDS) demonstrated that the samples were divided according to site and this grouping appeared to be significant according to ANOSIM and PERMANOVA analyses (*p* < 0.001) (**Figure 4A**). Notably, grouping according to season was not significant (*p* > 0.05). CCA, a method of constrained ordination (i.e., showing which environmental variables explain observed differences in community composition), showed that site together with P_ca_, pH, and TOC were significant environmental factors shaping the structure of bacterial communities in analyzed samples (**Figure 4B**). Percent variance explained by the variables found in the CCA analysis was assessed with variance partitioning. Total variance explained by the variable in question is followed by variance explained solely by this variable: site – 25.00% (7.00%), P_ca_ – 15.60% (0.10%), pH – 14.40% (3.20%), TOC – 9.30% (1.00%).

Fungal communities, like bacterial ones, were grouped according to test site, and the grouping was significant (ANOSIM and PERMANOVA analyses, *p* < 0.001) (**Figure 4C**). Similarly to bacterial communities, CCA analysis revealed significant effect of site, P_ca_ and pH (**Figure 4D**). Variance partitioning showed that season site explained 7.90% (3.70%), SP – 6.10% (3.90%), P_ca_ – 5.80% (0.70%), pH – 5.10% (4.30%) of variance.

### Functional Analysis of Bacterial and Fungal Communities

**FIGURE 5:**
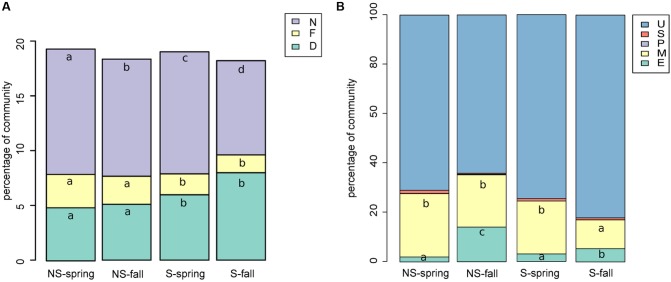
Functional classification of **(A)** bacterial sequences: shares of bacterial functional groups potentially involved in nitrogen cycling, D, denitrifying; N, nitrifying; NF, nitrogen-fixing **(A)**; **(B)** fungal sequences, E, endophytic; M, mycorrhizae forming; P, parasitic/pathogenic; S, saprophytic; U, unknown/unclassifiable **(B)**. Classification results were added to the OTU table and graphs were prepared and Kruskal–Wallis tests were performed.

Bacterial sequences were classified to functional groups potentially involved in nitrogen cycling based on their similarity to 16S rRNA genes of organisms of known function (**Figure 5A**). Sequences similar to those coming from denitrifiers were more frequent at the S site, while nitrifying and nitrogen-fixing ones were more abundant at the NS site. Counter intuitively, the potentially halotolerant taxa were more frequent at the NS site in spring (data not shown).

Fungal sequences were classified based on descriptions of species found in NCBI’s databases (**Figure 5B**). The percentage of saprophytic and parasitic/pathogenic taxa appeared to be consistently low. Occurence of endophytes and mycorrhizal fungi turned out to be negatively correlated, although the correlation was not significant (Spearman’s ρ = -0.14, *p*-value = 0.2351). Endophytes were more frequent in fall and at the NS site, while the opposite was true for mycorrhizal fungi.

### PICRUSt Analysis

PCA analysis demonstrated that sets of KOs were different in genomes of organisms coming from the two investigated sites (Supplementary Figure 4).

**FIGURE 6:**
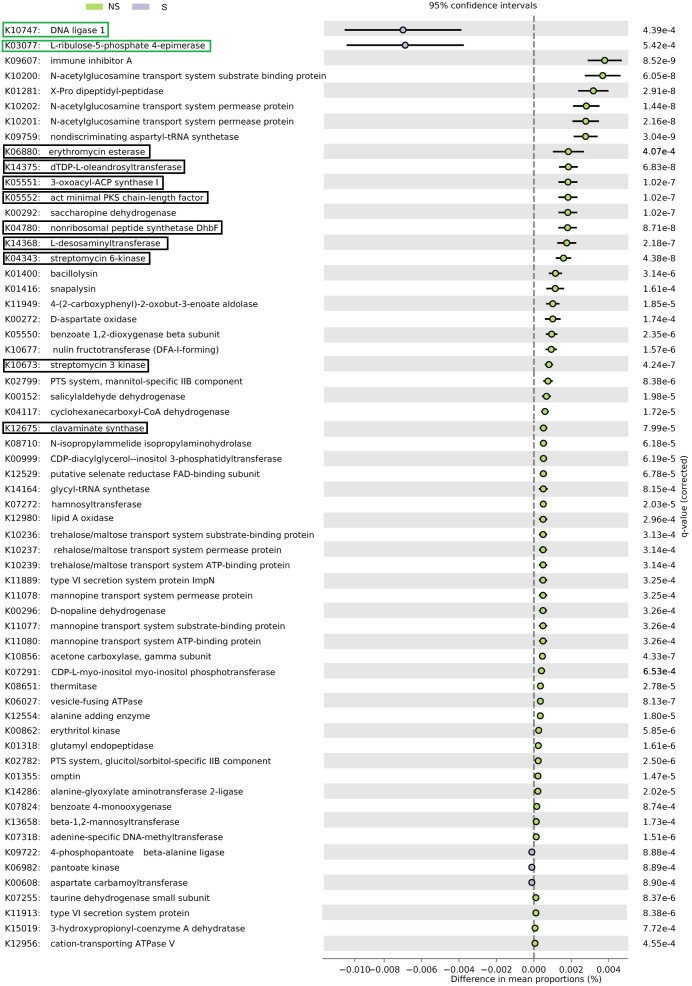
PICRUSt analysis of bacterial sequences. Barplots for most significantly different KO categories. Error bars represent standard error of the mean of given category abundance in different sample types. Categories belonging to DNA replication and repair supercategory and are shown in green boxes, while antibiotic synthesis and resistance is displayed in black boxes. *Q*-values (False Discovery Rates, based on two-sided Welche’s test with Benjamini-Hochberg correction) are given to the right of each category plot.

Altogether, 5101 KO categories were found by PICRUSt to be encoded in genomes of organisms thriving in alder root samples. Functional diversity (measured as the number of categories) did not differ significantly between samples groups, nor between sites (Kruskal–Wallis test, *p* > 0.05, Supplementary Table 1). Sixty-six KO categories were significant (**Figure 6**), five of them were represented more frequently in genomes of organisms coming from the S site. They were responsible for DNA replication and repair, biosynthesis and metabolism of pyrimidines as well as CoA biosynthesis. The remaining 61 categories were more frequent at the NS site. They were engaged in a plethora of processes, such as amino acids, peptides, carbohydrates, opines, and aromatic hydrocarbons metabolism, as well as type IV secretion, resistance to antibiotics and their biosynthesis (Supplementary Table 3).

## Discussion

Each plant species hosts a genotype-specific microbiome (i.e., endophytic microbiome) that dynamically responds to the environment, e.g., soil quality (Podolich et al., 2015). A recent meta-analysis of soil microbial communities revealed that the global microbial composition in saline soils is affected more by salinity than by extremes of any other abiotic factor, e.g., pH or temperature (Lozupone and Knight, 2007). There is a direct relationship between soil and root salinity levels, which may significantly influence endophytic microbial community structure (Yaish et al., 2016b). However, the effect of salinity on endophytic communities is largely unexplored. Most of the studies on the endophytes were focused on non-woody crops, moreover most of them used culture-dependent methods (Shen and Fulthorpe, 2015; Szymańska et al., 2016). Data on bacterial and fungal endophytes in roots of forest trees under saline stress are very limited (Ju et al., 2014; Hrynkiewicz et al., 2015; Thiem et al., 2017), and our work is the first report describing application of metagenomics to such a community with particular emphasis on root symbionts.

In general, we have noted more bacterial than fungal OTUs in alder roots, which may be caused by the 10-fold greater number of bacterial than fungal species observed in most soils (Larsen et al., 2017). Consistently bacteria showed higher diversity (measured as Shannon’s H’) than fungi. Moreover, our findings demonstrated significant differences in the endophytic microbial community composition due to the level of salt in the soil. Bacterial diversity, species richness, and evenness of black alder roots was decreased at the saline site (S), an effect similar to that observed by Yaish et al. (2016a) in *Phoenix dactylifera*. The lower average number of OTUs observed at the S site can be due to the fact that only a fraction of endophytes can thrive under conditions of increased salinity.

Black alder roots endophytic bacterial community was dominated by three phyla: Proteobacteria, Actinobacteria, and Acidobacteria that are the most common groups found in studies concerning soils (e.g., Gołębiewski et al., 2014; Canfora et al., 2017), including saline ones (Valenzuela-Encinas et al., 2009; Ma and Gong, 2013). Our results are also in agreement with studies of Shakya et al. (2013) concerning root endophytes of *Populus deltoides*. Moreover, our studies showed that Alphaproteobacteria, Actinobacteria, and Betaproteobacteria were found more frequently at the NS site, which is consistent with report of Yaish et al. (2016b). Decrease of Alphaproteobacteria and Actinobacteria level in response to environmental stress is a common observation in soil studies (e.g., Chodak et al., 2013; Gołębiewski et al., 2014). In our study, Acidobacteria and Gammaproteobacteria were more frequently found at the S site. It can be related to the higher abundance of this bacteria in saline soils (e.g., Yang et al., 2016; Yaish et al., 2016b).

Bacteria belonging to *Rhodanobacter*, *Granulicella*, and *Sphingomonas* genera were significantly more abundant in libraries coming from black alder roots from the S site. *Rhodanobacter* is a Gram-negative, aerobic bacterium currently not regarded as halotolerant. However, this bacterial species was found as a relatively abundant organism in *A. glutinosa* nodules (McEwan et al., 2017). *Sphingomonas* sp. belongs to a group of Gram-negative, chemoheterotrophic, aerobic bacteria that are widely distributed in nature, having been isolated from many different land and water habitats as well as from plant roots (Yang et al., 2016). Representatives of this genus make up a significant proportion of endophytic community in many trees (Izumi et al., 2008). They have the capacity to survive at low nutrient concentrations, as well as to metabolize a wide variety of carbon sources and some of them showed characteristics of nitrogen fixation and denitrification (Yang et al., 2016). Moreover, different species of *Sphingomonas* were shown to be endophytes effective in protection of crops against salts stress (Khan et al., 2014, 2017). Members of the *Granulicella* genus were found in the nodules of alder, similarly to *Rhodanobacter* (McEwan et al., 2017). Interestingly, according to Marupakula (2016), *Rhodanobacter* and *Granulicella* belong to bacteria colonizing ectomycorrhiza in boreal forest, which may suggest that they are mycorrhiza helper bacteria.

*Frankia* was found to be scarce in our study, and was found exclusively at the saline site. This might have been caused by a specific spatial distribution of these bacteria in plant roots, namely they are found mainly in nodules (Diagne et al., 2013). Roy et al. (2017) found 12 *Frankia* OTUs in *A. glutinosa* nodules, however, the bacteria might constitute <1% of all sequences (McEwan et al., 2017), which is similar to our results. This scarcity might have been caused by bias imposed by universal primers used. The universal primers were used to obtain broad picture of the bacterial community, at the expense of underrepresentation of certain groups.

*Bradyrhizobium* and *Rhizobium* were more abundant at the NS site. Members of both of these bacterial genera are common microsymbionts of nodulating legumes (Guimarães et al., 2015). However, many data indicate that non-legumes also react to the presence of bradyrhizobia and rhizobia in the rhizosphere. Root hair curling induced by these symbiotic bacteria was observed on maize, rice and oat plants (Antoun et al., 1998). To date, *Bradyrhizobium* was found to be endosymbiont of only one tree species – *Parasponia* (Trinick and Hadobas, 1988). *Bradyrhizobium* sp. is relatively sensitive to unfavorable environmental conditions, e.g., salinity (it tolerates up to 0.5–1% NaCl) (Guimarães et al., 2015) or soil pH (Rascovan et al., 2016). A member of the *Rhizobium* genus (*R. metallidurans*) was isolated from roots of silver birch and alder growing on heavy metals-contaminated sites (Złoch et al., 2016). However, representative of these genera can be also robust heterotrophs that can persist in bulk soil (Gano-Cohen et al., 2016).

Classification of bacterial OTUs into functional groups indicated that sequences from those potentially involved in nitrogen cycling comprised together ∼20% of their total number. This high percentage is in accordance with the key role of bacteria in nitrogen cycling in soil (Boyle et al., 2008). However, it must be noted that such a kind of analysis is intrinsically limited, as there may exist organisms with closely related 16S rRNA sequences, yet differing in genome content and not performing the same ecological roles. Therefore, these results should be treated with caution. Nitrogen-fixing bacteria were significantly more frequent at the NS site, and these organisms belonged to Alphaproteobacteria (*Rhizobium* and *Bradyrhizobium* genera). It might be connected to possible greater abundance of these taxa in non-saline soils, as it is known that environmental stress decreases level of Alphaproteobacteria in soils (e.g., Chodak et al., 2013).

To learn more on possible functions contained in genomes of organisms found in our samples, PICRUSt analysis was performed. Possible gene content was imputed based on Greengenes classification of OTUs. This approach allows to get insight into metagenomes without performing shotgun sequencing, which is much more expensive than amplicon sequencing. However, it has its intrinsic limits, due to the lack of certain sequences in the database and the fact that even organisms closely related in terms of 16S rRNA sequences may significantly differ when it comes to the gene content. Moreover, one has to bear in mind that this kind of analysis yields potential functions that does not need to be expressed. Nevertheless, when carefully interpreted, PICRUSt may yield interesting results at a very low cost. Our results suggest that bacterial functional diversity does not differ between sites, and, as antibiotic biosynthesis and resistance genes were more frequent at the site, that competition is more intensive. This is supported by the greater diversity observed at the NS site. On the other hand, higher salinity at the S site might have selected organisms more adapted to extreme environments requiring greater capabilities of DNA repair.

The ITS region sequencing indicated a positive relationship between higher salinity and biodiversity. We suggest that this phenomenon can be caused by two mechanisms: (i) salt-sensitive fungi may more readily enter plant roots where the environment is more stable, and (ii) plants may attract endophytes under stress conditions, as they might increase their tolerance to the stressors.

These mechanisms are connected with plant reaction to abiotic stress. Differences in the outside environment put the plant metabolism out of homeostasis and impose the necessity to harbor specific genetic and metabolic traits within its cellular system (Meena et al., 2017). Plant microbiome often facilitates reduction of abiotic stress (Singh et al., 2011; Meena et al., 2017), and endophytes from different habitats confer habitat-specific stress tolerance to plants (Rodriguez et al., 2004). It is also known that plant physiology together with environmental physico-chemical parameters determine endophytic community structure (Hardoim et al., 2015).

Alder’s endophytic fungal community was dominated by members of Agaricomycetes, Leotiomycetes, and Mortierellomycotina Incertae Sedis. In spite of large differences in taxa abundances between sites and seasons, statistical analysis indicated that the differences were not significant. This effect was caused by large variability among analyzed replicates, which might have been caused by specific spatial distribution of fungal mycelia, among them the mycorrhizal ones. We observed lower abundance of Agaricomycetes and Leotiomycetes at the NS site in spring. These groups comprise many ectomycorrhizal representatives that may be less abundant under conditions of high soil phosphorus content (Balzergue et al., 2013; Nouri et al., 2014). Our analysis revealed mainly ectomycorrhizal fungi, while arbuscular ones turned out to be rare in our dataset. This is probably due to two factors: (i) primers’ specificity and (ii) the age of the tree stands under study. Arbuscular mycorrhizal structures usually are formed only at early stages of seedling development (van der Heijden, 2001; Põlme et al., 2013).

*Tomentella*, *Lactarius*, and *Phialocephala* members were more abundant at the S site. *Tomentella* was commonly found as an ectomycorrhizal partner of many trees, such as willow, birch, or alder, with tendency to dominate at unfavorable environmental conditions (Ishida et al., 2009; Regvar et al., 2010; Hrynkiewicz et al., 2015). Our results suggest that members of this genus can be tolerant to salinity. Mechanisms involved in *Tomentella* tolerance to abiotic environmental stressors still remain to be elucidated. *Lactarius*, belonging to russula-lactarius fungal linkages (Tedersoo et al., 2009), was frequently identified in alder roots (Kennedy and Hill, 2010; Rochet et al., 2011), but never in saline environment. *Phialocephala* spp. belong to dark septate endophytes (DSE) and dominate the endophytic mycobiota in roots of conifers and members of the Ericaceae family in heathlands, forests, and alpine ecosystems (Grünig et al., 2009). *Phialocephala* spp. may form mycorrhizal structures or live as endophytes (Lukešová et al., 2015).

Contrary to the above-mentioned fungal taxa, *Mortierella* and *Naucoria* were found more frequently at the NS site. Members of the *Mortierella* genus can grow on a wide variety of substrates, including chitin (Leake and Read, 1990), and are commonly found as soil-inhabiting saprophytes (Kirk et al., 2008), however, some species were found to adopt endophytic lifestyle (Jankowiak et al., 2016). They were also found to increase effectiveness of mycorrhiza formation and to solubilize phosphorus (Zhang et al., 2011). This fungal genus is known to prefer high organic matter content (Wagner et al., 2013) and its abundance was greater at the non-saline site. It is thus possible that the higher level of extractable phosphorus at the NS site is related to the incidence of *Mortierella*. Genus *Naucoria* (also known as *Alnicola*) comprises many closely related endophytic species that colonize *Alnus* roots (e.g., Moreau et al., 2006; Rochet et al., 2011).

As salinity and other soil physico-chemical parameters differed significantly between the test sites, we expected that the identity of sites would be the key driver of microbial diversity. Indeed, unconstrained ordinations showed that the samples coming from the same site clustered together, moreover, this grouping appeared to be significant. This was corroborated by the CCA analysis, wherein site TOC, pH, parasitic/pathogenic (P), and SP were found to be significant factors explaining variance in microbial communities. Seasonality is commonly regarded as an important parameter influencing plant-associated microbial communities (Hrynkiewicz et al., 2015; Shen and Fulthorpe, 2015). This is due to the differences in nutrients levels and temperature, both factors can change microorganisms’ abundance and community structure (Carrero-Colo et al., 2006), however, we did not see significant influence of season on the communities under study. This effect might be explained by relative stability of environment within plants. Moreover, as we analyzed the communities by amplification of rDNA, we were unable to capture changes in microorganismal activity that probably fluctuated seasonally.

## Conclusion

Salinity affects both bacterial and fungal diversity, albeit in different manners. Bacterial diversity decreases with salinity, while the response of fungi to this parameter is more complex. Bacterial and fungal community structure depends on salinity, and seasonality does not appear to be an important factor explaining variance in communities of root alder endophytes. There are taxa that are more abundant at the saline site: *Rhodanobacter*, *Sphingomonas*, and *Granulicella* (bacteria); *Tomentella*, *Lactarius*, *Phialocephala* (fungi). We found a low number of sequences coming from obvious halotolerant/halophilic bacteria and fungi in our dataset. Ectomycorrhizal fungi (*Tomentella*, *Lactarius*, and *Thelephora*) are an important component of endophytic community of alder roots at the saline site. The lasting challenge would be to evaluate microbiome influence on nutrients cycling and plant physiology of alder at a greater number of sites differing in salinity. This could be done by means of shotgun metatranscriptomic analysis coupled with marker amplicons sequencing both at the DNA and RNA levels.

## Author Contributions

DT did all the laboratory analyses and wrote the first version of the manuscript. MG designed the sequencing system, managed the lab experiments, performed the bioinformatic and statistical analyses, and participated in the preparation of the manuscript. PH did the soil chemical analyses and interpreted the results. AP selected the areas for field experiments, analyzed the M level in soils, and interpreted the results of soil analysis. KH designed and managed the field and lab experiments as well as participated in the preparation of the manuscript. All authors revised the manuscript and approved the publication.

## Conflict of Interest Statement

The authors declare that the research was conducted in the absence of any commercial or financial relationships that could be construed as a potential conflict of interest.

## Footnotes

1 https://github.com/rprops/PICRUSt_from_mothur

2 www.genome.jp

## References

[B1] Antoun H. Beauchamp C. J. Goussard N. Chabot R. Lalande R. (1998). Potential of *Rhizobium* and *Bradyrhizobium* species as plant growth promoting rhizobacteria on non-legumes: effects on radish (*Raphanus sativus* L.). *Plant Soil* 204 57–67. 10.1023/A:1004326910584

[B2] Asghari H. R. Marschner P. Smith S. E. Smith F. A. (2005). Growth response of *Atriplex nummularia* to inoculation with arbuscular mycorrhizal fungi at different salinity levels. *Plant Soil* 373 245–256. 10.1007/s11104-004-7942-6

[B3] Balzergue C. Chabaud M. Barker D. G. Bécard G. Rochange S. F. (2013). High phosphate reduces host ability to develop arbuscular mycorrhizal symbiosis without affecting root calcium spiking responses to the fungus. *Front. Plant Sci.* 29:426. 10.3389/fpls.2013.00426 24194742 PMC3810610

[B4] Bencherif K. Boutekrabt A. Fontaine J. Laruelle F. Dalpè Y. Sahraoui A. (2015). Science of the total environment impact of soil salinity on arbuscular mycorrhizal fungi biodiversity and microflora biomass associated with *Tamarix articulata* Vahll rhizosphere in arid and semi-arid Algerian areas. *Sci. Total Environ.* 533 488–494. 10.1016/j.scitotenv.2015.07.007 26184906

[B5] Bengtsson-Palme J. Veldre V. Ryberg M. Hartmann M. Branco S. Wang Z. (2013). ITSx: improved software detection and extraction of ITS1 and ITS2 from ribosomal ITS sequences of fungi and other eukaryotes for use in environmental sequencing. *Methods Ecol. Evol.* 4 914–919. 10.1111/2041-210X.12073

[B6] Boyle S. A. Yarwooda R. R. Bottomley P. J. Myrolda D. D. (2008). Bacterial and fungal contributions to soil nitrogen cycling under Douglas fir and red alder at two sites in Oregon. *Soil Biol. Biochem.* 40 443–451. 10.1016/j.soilbio.2007.09.007

[B7] Canfora L. Salvati L. Benedetti A. Francaviglia R. (2017). Is soil microbial diversity affected by soil and groundwater salinity? Evidences from a coastal system in central Italy. *Environ. Monit. Assess.* 189:319. 10.1007/s10661-017-6040-1 28589460

[B8] Carrero-Colo M. Nakatsu C. H. Konopka A. (2006). Effect of nutrient periodicity on microbial community dynamics. *Appl. Environ. Microbiol.* 72 3175–3183. 10.1128/AEM.72.5.3175-3183.2006 16672455 PMC1472307

[B9] Chandrasekaran M. Boughattas S. Hu S. Oh S. H. Sa T. (2014). A meta-analysis of arbuscular mycorrhizal effects on plants grown under salt stress. *Mycorrhiza* 24 611–625. 10.1007/s00572-014-0582-7 24770494

[B10] Chen D. M. Ellul S. Herdman K. Cairney J. W. G. (2001). Influence of salinity on biomass production by Australian *Pisolithus* spp. isolates. *Mycorrhiza* 11 231–236. 10.1007/s005720100126

[B11] Chen S. Hawighorst P. Sun J. Polle A. (2014). Salt tolerance in *Populus*: significance of stress signaling networks, mycorrhization, and soil amendments for cellular and whole-plant nutrition. *Environ. Exp. Bot.* 107 113–124. 10.1016/j.envexpbot.2014.06.001

[B12] Chodak M. Gołębiewski M. Morawska-Płoskonka K. Kudak K. Niklińska M. (2013). Diversity of microorganisms from forest soils differently polluted with heavy metals. *Appl. Soil Ecol.* 64 7–14. 10.1016/j.apsoil.2012.11.004

[B13] Dadlez R. Jaroszewski W. (1994). *Tectonics.* Warsaw: PWN.

[B14] Deja-Sikora E. (2012). *Search for Bacterial Cadmium, Zinc, Lead, Copper and Chromium Resistance Genes in Metagenome of Soils Polluted with Heavy Metals.* Ph.D. thesis, Nicolaus Copernicus University, Torun.

[B15] DeSantis T. Z. Hugenholtz P. Larsen N. Rojas M. Brodie E. L. Keller K. (2006). Greengens, a chimera-checked 16S rRNA gene database and workbench compatible with ARB. *Appl. Environ. Microbiol.* 72 5069–5072. 10.1128/AEM.03006-05 16820507 PMC1489311

[B16] Diagne N. Arumugama K. Ngom M. Nambiar-Veetil M. Franche C. Narayanan K. K. (2013). Use of *Frankia* and actinorhizal plants for degraded lands reclamation. *BioMed Res. Int.* 2013:948258. 10.1155/2013/948258 24350296 PMC3844217

[B17] Dixon R. K. Rao M. V. Garg V. K. (1993). Salt stress affects *in vitro* growth and *in situ* symbioses of ectomycorrhizal fungi. *Mycorrhiza* 3 63–68. 10.1007/BF00210694

[B18] Edgar R. C. Haas B. J. Clemente J. C. Quince C. Knight R. (2011). UCHIME improves sensitivity and speed of chimera detection. *Bioinformatics* 27 2194–2200. 10.1093/bioinformatics/btr381 21700674 PMC3150044

[B19] Estrada B. Aroca R. Maathuis J. M. Barea J. M. Ruiz-Lozano J. M. (2013). Arbuscular mycorrhizal fungi native from a Mediterranean saline area enhance maize tolerance to salinity through improved ion homeostasis. *Plant Cell Environ.* 36 1771–1782. 10.1111/pce.12082 23421735

[B20] Evelin H. Giri B. Kapoor R. (2012). Contribution of *Glomus intraradices* inoculation to nutrient acquisition and mitigation of ionic imbalance in NaCl-stressed *Trigonella foenum-graecum*. *Mycorrhiza* 22 203–217. 10.1007/s00572-011-0392-0 21695577

[B21] Evelin H. Kapoor R. (2013). Arbuscular mycorrhizal symbiosis modulates antioxidant response in salt-stressed *Trigonella foenum-graecum* plants. *Mycorrhiza* 24 197–208. 10.1007/s00572-013-0529-4 24113907

[B22] Gano-Cohen K. A. Stokes P. J. Blanton M. A. Wendlandt C. E. Hollowell A. C. Regus J. U. (2016). Nonnodulating *Bradyrhizobium* spp. modulate the benefits of legume-*Rhizobium* mutualism. *Appl. Environ. Microbiol.* 1 5259–5268. 10.1128/AEM.01116-16 27316960 PMC4988196

[B23] Gołębiewski M. Deja-Sikora E. Cichosz M. Tretyn A. Wróbel B. (2014). 16S rDNA pyrosequencing analysis of bacterial community in heavy metals polluted soils. *Microb. Ecol.* 67 635–647. 10.1007/s00248-013-0344-7 24402360 PMC3962847

[B24] Grünig C. R. Queloz V. Duò A. Sieber T. N. (2009). Phylogeny of *Phaeomollisia piceae* gen. sp. nov.: a dark, septate, conifer-needle endophyte and its relationships to *Phialocephala* and *Acephala*. *Mycol. Res.* 113 207–221. 10.1016/j.mycres.2008.10.005 19015028

[B25] Guimarães A. A. Florentinoa L. A. Almeidaa K. A. Lebbeb L. Silvaa K. B. Willemsb A. (2015). High diversity of *Bradyrhizobium* strains isolated from several legume species and land uses in Brazilian tropical ecosystems. *Syst. Appl. Microbiol.* 38 433–441. 10.1016/j.syapm.2015.06.006 26234199

[B26] Hajiboland R. Aliasgharzadeh N. Laiegh S. F. Poschenreider C. (2010). Colonization with arbuscular mycorrhizal fungi improves salinity tolerance of tomato (*Solanum lycopersicum* L.) plants. *Plant Soil* 331 313–327. 10.1007/s11104-009-0255-z

[B27] Hameed A. Dilfuza E. Abd-Allah E. F. Hashem A. Kumar A. Ahmad P. (2014). “Salinity stress and arbuscular mycorrhizal symbiosis in plants,” in *Use of Microbes for the Alleviation of Soil Stresses*, ed. Miransari M. (New York, NY: Springer), 139–159.

[B28] Hardoim P. R. van Overbeek L. S. Berg G. Pirttilä A. M. Compant S. Campisano A. (2015). The Hidden world within plants: ecological and evolutionary considerations for defining functioning of microbial endophytes. *Microbiol. Mol. Biol. Rev.* 79 293–320. 10.1128/MMBR.00050-14 26136581 PMC4488371

[B29] Hrynkiewicz K. Szymańska S. Piernik A. Thiem D. (2015). Ectomycorrhizal community structure of *Salix* and *Betula* spp. at a saline site in central Poland in relation to the seasons and soil parameters. *Water Air Soil Pollut.* 226:99. 10.1007/s11270-015-2308-7 25821257 PMC4365278

[B30] Ishida T. A. Nara K. Ma S. Takano T. Liu S. (2009). Ectomycorrhizal fungal community in alkaline-saline soil in northeastern China. *Mycorrhiza* 19 329–335. 10.1007/s00572-008-0219-9 19104846

[B31] Izumi H. Cairney J. W. Killham K. Moore E. Alexander I. J. Anderson I. C. (2008). Bacteria associated with ectomycorrhizas of slash pine (*Pinus elliottii*) in south-eastern Queensland, Australia. *FEMS Microbiol. Lett.* 282 196–204. 10.1111/j.1574-6968.2008.01122.x 18355286

[B32] Jankowiak R. Bilański P. Paluch J. Kołodziej Z. (2016). Fungi associated with dieback of *Abies alba* seedlings in naturally regenerating forest ecosystems. *Fungal Ecol.* 24 61–69. 10.1016/j.funeco.2016.08.13

[B33] Joshi N. A. Fass J. N. (2011). *Sickle: A Sliding-window, Adaptive, Quality-based Trimming Tool for FastQ Files (Version 1.33) [Software].* Available at: https://github.com/najoshi/sickle

[B34] Ju X. Ouyang L. Zhang L. (2014). Endophytes community of *Populus euphratica* Oliv and the strains improving the salt tolerance of plant. *J. Pure Appl. Microbiol.* 8 43–51.

[B35] Kennedy P. G. Hill L. T. (2010). A molecular and phylogenetic analysis of the structure and specificity of *Alnus rubra* ectomycorrhizal assemblages. *Fungal Ecol.* 3 195–204. 10.1016/j.funeco.2009.08.005

[B36] Khan A. L. Waqas M. Asaf S. Kamran M. Shahzad R. Bilal S. (2017). Plant growth-promoting endophyte *Sphingomonas* sp. LK11 alleviates salinity stress in *Solanum pimpinellifolium*. *Environ. Exp. Bot.* 133 58–69. 10.1016/j.envexpbot.2016.09.009

[B37] Khan A. L. Waqas M. Kang S. M. Al-Harrasi A. Hussain J. Al-Rawahi A. (2014). Bacterial endophyte *Sphingomonas* sp. LK11 produces gibberellins and IAA and promotes tomato plant growth. *J. Microbiol.* 52 689–695. 10.1007/s12275-014-4002-7 24994010

[B38] Kirk P. M. Cannon P. F. Minter D. W. Stalpers J. A. (2008). *Dictionary of the Fungi*, 10th Edn Wallingford: CAB International.

[B39] Langenfeld-Heyser R. Gao J. Ducic T. Tachd P. Lu C. F. Fritz E. (2007). *Paxillus involutus* mycorrhiza attenuate NaCl-stress responses in the salt-sensitive hybrid poplar *Populus × canescens*. *Mycorrhiza* 17 121–131. 10.1007/s00572-006-0084-3 17115201

[B40] Langille M. G. I. Zaneveld J. Caporaso J. G. McDonald D. Knights D. A Reyes J. (2013). Predictive functional profiling of microbial communities using 16S rRNA marker gene sequences. *Nat. Biotechnol.* 31 814–821. 10.1038/nbt.2676 23975157 PMC3819121

[B41] Larsen B. B. Miller E. C. Rhodes M. K. Wiens J. J. (2017). Inordinate fondness multiplied and redistributed: the number of species on earth and the new pie of life. *Q. Rev. Biol.* 92 229–265. 10.1086/693564

[B42] Leake J. R. Read D. J. (1990). Chitin as a nitrogen source for mycorrhizal fungi. *Mycol. Res.* 94 993–995. 10.1016/S0953-7562(09)81318-X 24386977

[B43] Lozupone C. A. Knight R. (2007). Global patterns in bacterial diversity. *Proc. Natl. Acad. Sci. U.S.A.* 104 11436–11440. 10.1073/pnas.0611525104 17592124 PMC2040916

[B44] Lukešová T. Kohout P. Větrovský T. Vohník M. (2015). The potential of dark septate endophytes to form root symbioses with ectomycorrhizal and ericoid mycorrhizal middle European forest plants. *PLoS One* 10:e0124752. 10.1371/journal.pone.0124752 25905493 PMC4408093

[B45] Ma B. Gong J. (2013). A meta-analysis of the publicly available bacterial and archaeal sequence diversity in saline soils. *World J. Microbiol. Biotechnol.* 29 2325–2334. 10.1007/s11274-013-1399-9 23756871

[B46] Manchanda G. Garg N. (2011). Alleviation of salt-induced ionic, osmotic and oxidative stresses in *Cajanus cajan* nodules by AM inoculation. *Plant Biosyst.* 145 88–97. 10.1080/11263504.2010.539851

[B47] Marupakula S. (2016). *Interactions between Ectomycorrhizal Associations and Bacteria.* Ph.D. thesis, University of Agricultural Sciences, Uppsala.

[B48] Masella A. P. Bartram A. K. Truszkowski J. M. Brown D. G. Neufeld J. D. (2012). PANDAseq: paired-end assembler for Illumina sequences. *BMC Bioinformatics* 14:31. 10.1186/1471-2105-13-31 22333067 PMC3471323

[B49] McEwan N. R. Wilkinson T. Girdwood S. E. Snelling T. J. Collins T. Dougal K. (2017). Evaluation of the microbiome of decaying alder nodules by next generation sequencing. *Endocyt. Cell Res.* 28 14–19.

[B50] Meena K. K. Sorty A. M. Bitla U. M. Choudhary K. Gupta P. Pareek A. (2017). Abiotic stress responses and microbe-mediated mitigation in plants: the Omics strategies. *Front. Plant Sci.* 9:172. 10.3389/fpls.2017.00172 28232845 PMC5299014

[B51] Meharg A. A. (2003). The mechanistic basis of interactions between mycorrhizal associations and toxic metal cations. *Mycol. Res.* 107 1253–1265. 10.1017/S0953756203008608 15000228

[B52] Moreau P. A. Peintner U. Gardes M. (2006). Phylogeny of the ectomycorrhizal mushroom genus *Alnicola* (Basidiomycota, Cortinariaceae) based on rDNA sequences with special emphasis on host specificity and morphological characters. *Mol. Phylogenet. Evol.* 38 794–807. 10.1016/j.ympev.2005.10.008 16314113

[B53] Neefs J. M. Van de Peer Y. De Rijk P. Chapelle S. De Wachter R. (1993). Compilation of small ribosomal subunit RNA structures. *Nucleic Acids Res.* 21 3025–3049. 10.1093/nar/21.13.3025 8332525 PMC309731

[B54] Ngom M. Oshone R. Diagne N. Cissoko M. Svistoonoff S. Tisa L. S. (2016). Tolerance to environmental stress by the nitrogen-fixing actinobacterium *Frankia* and its role in actinorhizal plants adaptation. *Symbiosis* 70 17–29. 10.1007/s13199-016-0396-9

[B55] Nikolenko S. I. Korobeynikov A. I. Alekseyev M. A. (2013). BayesHammer: Bayesian clustering for error correction in single-cell sequencing. *BMC Genomics* 14(Suppl. 1):S7. 10.1186/1471-2164-14-S1-S7 23368723 PMC3549815

[B56] Nouri E. Breuillin-Sessoms F. Feller U. Reinhardt D. (2014). Phosphorus and nitrogen regulate arbuscular mycorrhizal symbiosis in *Petunia hybrida*. *PLoS One* 9:e90841. 10.1371/journal.pone.0090841 24608923 PMC3946601

[B57] Oliveira R. S. Castro P. M. L. Dodd J. C. Vosátka M. (2005). Synergistic effect of *Glomus intraradices* and *Frankia* spp. on the growth and stress recovery of *Alnus glutinosa* in an alkaline anthropogenic sediment. *Chemosphere* 60 1462–1470. 10.1016/j.chemosphere.2005.01.038 16054916

[B58] Oshone R. Mansour S. R. Tisa L. S. (2013). Effect of salt stress on the physiology of *Frankia* sp. strain CcI6. *J. Biosci.* 38 699–702. 10.1007/s12038-013-9371-2 24287648

[B59] Parks D. H. Tyson G. W. Hugenholtz P. Beiko R. G. (2014). STAMP: statistical analysis of taxonomic and functional profiles. *Bioinformatics* 30 3123–3124. 10.1093/bioinformatics/btu494 25061070 PMC4609014

[B60] Podolich O. Ardanov P. Zaets I. Pirttilä A. M. Kozyrovska N. (2015). Reviving of the endophytic bacterial community as a putative mechanism of plant resistance. *Plant Soil* 388 367–377. 10.1007/s11104-014-2235-1

[B61] Põlme S. Bahram M. Yamanaka T. Nara K. Dai Y. C. Grebenc T. (2013). Biogeography of ectomycorrhizal fungi associated with alders (*Alnus* spp.) in relation to biotic and abiotic variables at the global scale. *New Phytol.* 198 1239–1249. 10.1111/nph.12170 23421531

[B62] Pritsch K. Boyle H. Munch J. C. Buscot F. (1997). Characterization and identification of black alder ectomycorrhizas by PCR/RFLP analyses of the rDNA internal transcribed spacer (ITS). *New Phytol.* 137 357–369. 10.1046/j.1469-8137.1997.00806.x 33863178

[B63] Rascovan N. Carbonetto B. Perrig D. Díaz M. Canciani W. Abalo M. (2016). Integrated analysis of root microbiomes of soybean and wheat from agricultural fields. *Sci. Rep.* 6:28084. 10.1038/srep28084 27312589 PMC4911569

[B64] Regvar M. Likar M. Piltaver A. Kugonic N. Smith J. E. (2010). Fungal community structure under goat willows (*Salix caprea* L.) growing at metal polluted site: the potential of screening in a model phytostabilisation study. *Plant Soil* 330 345–356. 10.1007/s11104-009-0207-7

[B65] Rochet J. Moreau P. A. Manzi S. Gardes M. (2011). Comparative phylogenies and host specialization in the alder ectomycorrhizal fungi *Alnicola*, *Alpova* and *Lactarius* (Basidiomycota) in Europe. *BMC Evol. Biol.* 11:40. 10.1186/1471-2148-11-40 21306639 PMC3045908

[B66] Rodriguez R. J. Redman R. S. Henson J. M. (2004). The role of fungal symbioses in the adaptation of plants to high stress environments. *Mitig. Adapt. Strat. Glob. Change* 9 261–272 10.1023/B:MITI.0000029922.31110.97

[B67] Rognes T. Flori T. Nichols B. Quince C. Mahe F. (2016). vSEARCH: a versatile open source tool for metagenomics. *PeerJ* 4:e2584. 10.7717/peerj.2584 27781170 PMC5075697

[B68] Roy M. Pozzi A. C. Gareil R. Nagati M. Manzi S. Nouioui I. (2017). Alder and the Golden Fleece: high diversity of *Frankia* and ectomycorrhizal fungi revealed from *Alnus glutinosa* subsp. *barbata* roots close to a Tertiary and glacial refugium. *PeerJ* 18:e3479. 10.7717/peerj.3479 28729950 PMC5518731

[B69] Roy S. Khasa D. P. Greer C. W. (2007). Combining alders, frankiae, and mycorrhizae for soil remediation and revegetation. *Can. J. Bot.* 85 237–251. 10.1139/B07-017

[B70] Ruotsalainen A. L. Markkola A. M. Kozlov M. V. (2009). Mycorrhizal colonisation of mountain birch (*Betula pubescens* ssp. *czerepanovii*) along three environmental gradients: does life in harsh environments alter plant-fungal relationships? *Environ. Monit. Assess.* 148 215–232. 10.1007/s10661-007-0152-y 18327653

[B71] Sarwat M. Hashem A. Ahanger M. A. Elsayed F. A. Alqarawi A. A. Alyemeni M. N. (2016). Mitigation of NaCl stress by arbuscular mycorrhizal fungi through the modulation of osmolytes, antioxidants and secondary metabolites in mustard (*Brassica juncea* L.) plants. *Front. Plant Sci.* 7:869. 10.3389/fpls.2016.00869 27458462 PMC4931734

[B72] Schützendübel A. Polle A. (2002). Plant responses to abiotic stresses: heavy metal induced oxidative stress and protection by mycorrhization. *J. Exp. Bot.* 53 1351–1365. 10.1093/jexbot/53.372.1351 11997381

[B73] Shakya M. Gottel N. Castro H. Yang Z. K. Gunter L. Labbé J. (2013). A multifactor analysis of fungal and bacterial community structure in the root microbiome of mature *Populus deltoides* trees. *PLoS One* 8:e76382. 10.1371/journal.pone.0076382 24146861 PMC3797799

[B74] Shen S. Y. Fulthorpe R. (2015). Seasonal variation of bacterial endophytes in urban trees. *Front. Microbiol.* 6:427. 10.3389/fmicb.2015.00427 26042095 PMC4437045

[B75] Singh L. P. Gill S. S. Tuteja N. (2011). Unraveling the role of fungal symbionts in plant abiotic stress tolerance. *Plant Signal. Behav.* 6 175–191. 10.4161/psb.6.2.14146 21512319 PMC3121976

[B76] StatSoft Inc. (2006). *Statistica for Windows (Version 7.1) [Software].* Available at: www.statsoft.com

[B77] Szymańska S. Płociniczak T. Piotrowska-Seget Z. Hrynkiewicz K. (2016). Endophytic and rhizosphere bacteria associated with the roots of the halophyte *Salicornia europaea* L. – community structure and metabolic potential. *Microbiol. Res.* 192 37–51. 10.1016/j.micres.2016.05.012 27664722

[B78] Talaat N. B. Shawky B. T. (2014). Protective effects of arbuscular mycorrhizal fungi on wheat (*Triticum aestivum* L.) plants exposed to salinity. *Environ. Exp. Bot.* 98 20–31. 10.1016/j.envexpbot.2013.10.005

[B79] Tang M. Sheng M. Chen H. Zhang F. F. (2009). *In vitro* salinity resistance of three ectomycorrhizal fungi. *Soil Biol. Biochem.* 41 948–953. 10.1016/j.soilbio.2008.12.007

[B80] Tedersoo L. Suvi T. Jairus T. Ostonen I. Põlme S. (2009). Revisiting ectomycorrhizal fungi of the genus *Alnus*: differential host specificity, diversity and determinants of the fungal community. *New Phytol.* 182 727–735. 10.1111/j.1469-8137.2009.02792.x 19320837

[B81] Thiem D. Piernik A. Hrynkiewicz K. (2017). Ectomycorrhizal and endophytic fungi associated with *Alnus glutinosa* growing in a saline area of central Poland. *Symbiosis* 1–12. 10.1007/s13199-017-0512-5 29674805 PMC5899101

[B82] Trinick M. J. Hadobas P. A. (1988). Biology of the *Parasponia-Bradyrhizobium* symbiosis. *Plant Soil* 110 177–185. 10.1007/BF02226797

[B83] Valenzuela-Encinas C. Neria-Gonzalez I. Alcantara-Hernandez R. J. Estrada-Alvarado I. Zavala-Diaz de la Serna F. J. Dendooven L. (2009). Changes in the bacterial populations of the highly alkaline saline soil of the former lake Texcoco (Mexico) following flooding. *Extremophiles* 13 609–621. 10.1007/s00792-009-0244-4 19387766

[B84] van der Heijden E. W. (2001). Differential benefits of arbuscular mycorrhizal and ectomycorrhizal infection in *Salix repens*. *Mycorrhiza* 10 185–193. 10.1007/s005720000077

[B85] van Reeuwijk L. P. (2006). *Procedures for Soil Analysis.* Wageningen: ISRIC.

[B86] Wagner L. Stielow B. Hoffmann K. Petkovits T. Papp T. Vágvölgyi C. (2013). A comprehensive molecular phylogeny of the *Mortierellales* (*Mortierellomycotina*) based on nuclear ribosomal DNA. *Persoonia* 30 77–93. 10.3767/003158513X666268 24027348 PMC3734968

[B87] Wang H. Fan W. Yu P. S. Han J. (2003). “Mining concept-drifting data streams using ensemble classifiers,” in *Proceedings of the 9th ACM International Conference on Knowledge Discovery and Data Mining* (Washington, DC: SIGKDD), 226–235. 10.1145/956750.956778

[B88] Yaish M. W. Al-Harrasi I. Alansari A. S. Al-Yahyai R. Glick B. R. (2016a). The use of high throughput DNA sequence analysis to assess the endophytic microbiome of date palm roots grown under different levels of salt stress. *Int. Microbiol.* 19 143–155. 10.2436/20.1501.01.272 28494084

[B89] Yaish M. W. Al-Lawati A. Jana G. A. Vishwas Patankar H. Glick B. R. (2016b). Impact of soil salinity on the structure of the bacterial endophytic community identified from the roots of Caliph medic (*Medicago truncatula*). *PLoS One* 11:e0159007. 10.1371/journal.pone.0159007 27391592 PMC4938511

[B90] Yan N. Marschner P. Cao W. Zuo C. Qin W. (2015). Influence of salinity and water content on soil microorganisms. *Int. Soil Water Conserv. Res.* 3 316–323. 10.1016/j.iswcr.2015.11.003

[B91] Yang H. Hu J. Long X. Liu Z. Renge Z. (2016). Salinity altered root distribution and increased diversity of bacterial communities in the rhizosphere soil of Jerusalem artichoke. *Sci. Rep.* 6:20687. 10.1038/srep20687 26852800 PMC4745076

[B92] Zhang H. Wu X. Li G. Qui P. (2011). Interactions between arbuscular mycorrhizal fungi and phosphate-solubilizing fungus (*Mortierella* sp.) and their effects on *Kostelelzkya virginica* growth and enzyme activities of rhizosphere and bulk soils at different salinities. *Biol. Fertil. Soils* 47 543–554. 10.1007/s00374-011-0563-3

[B93] Złoch M. Thiem D. Gadzała-Kopciuch R. Hrynkiewicz K. (2016). Synthesis of siderophores by plant-associated metallotolerant bacteria under exposure to (Cd^2+^). *Chemosphere* 156 312–325. 10.1016/j.chemosphere.2016.04.130 27183333

