# Supplementary material for: How Does Salinity Shape Bacterial and Fungal Microbiomes of Alnus glutinosa Roots?
Source: Front Microbiol. 2018 Apr 18;9:651. doi: 10.3389/fmicb.2018.00651 (PMC5915629; doi:10.3389/fmicb.2018.00651)
Supplement: Supplementary file 1 [file Data_Sheet_1.docx]

Supplementary Material

How does salinity shape bacterial and fungal microbiomes of *Alnus glutinosa* roots?

**Dominika Thiem^1,2^, Marcin Gołębiewski^2,3^, Piotr Hulisz^4^, Agnieszka Piernik^5^, Katarzyna Hrynkiewicz^1,2^**

^1^ Department of Microbiology, Faculty of Biology and Environmental Protection,

Nicolaus Copernicus University, Toruń, Poland, ^*^ hrynk@umk.pl

^2^ Chair of Plant Physiology and Biotechnology, Faculty of Biology and Environmental Protection, NCU, Toruń, Poland,

^3^ Centre of Modern Interdisciplinary Technologies, NCU, Toruń, Poland

^4^ Department of Soil Science and Landscape Management, Faculty of Earth Sciences, NCU, Toruń, Poland

^5^ Chair of Geobotany and Landscape Planning, Faculty of Biology and Environmental Protection, NCU, Toruń, Poland

*** Correspondence:**

Katarzyna Hrynkiewicz

Phone: (+48) 56 61 14 447

Fax: (+48) 56 61 14 772

E-mail: hrynk@umk.pl, hrynkiewicz.katarzyna@gmail.com


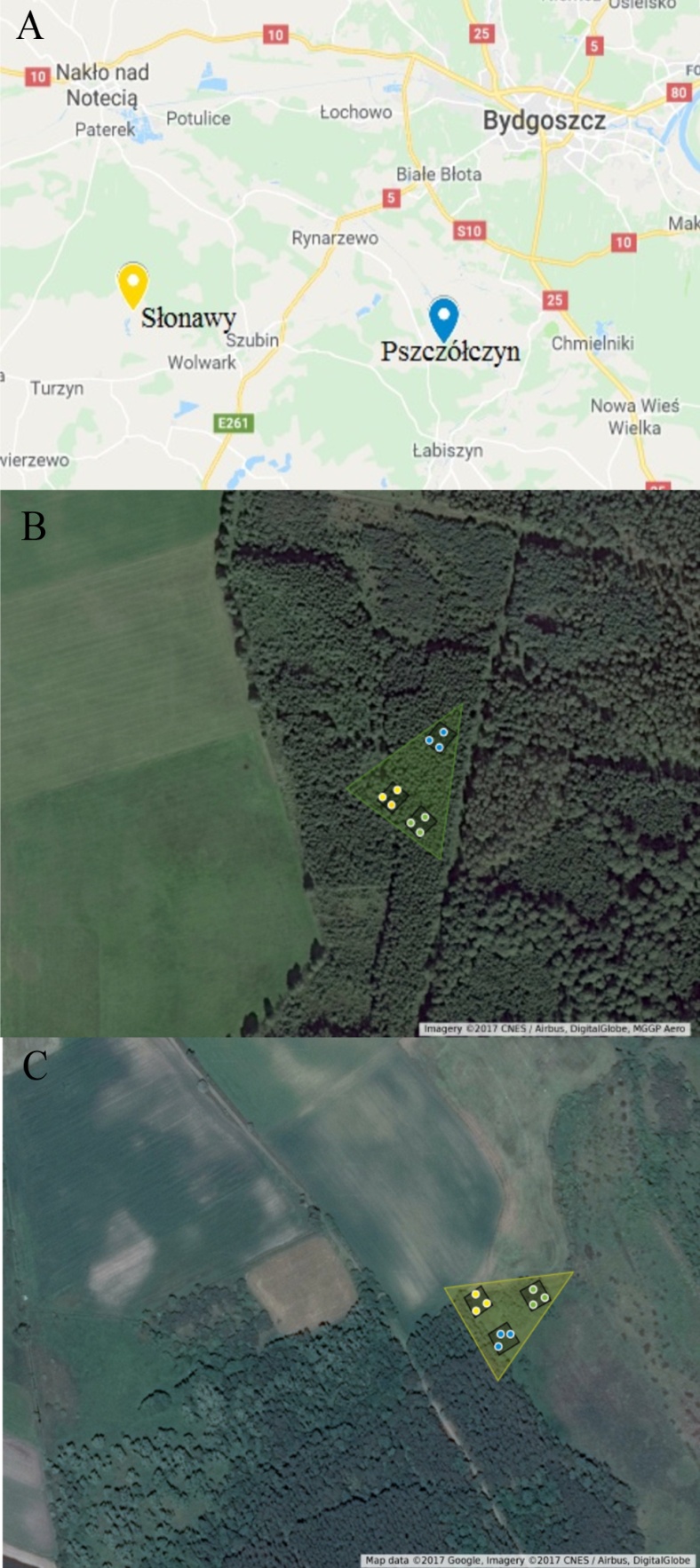


**Supplementary Figure 1.** Test sites: (A) location in the country, (B) plots in the non-saline site in Pszczółczyn, (C) plots in saline site in Słonawy.

**Materials and methods**

**Methodology of bacterial sequences qualification to functional groups.**

First, relevant sequences were retrieved from NCBI's NR database on Dec. 17, 2017 with the following queries: for nitrogen-fixing bacteria – (bacteria[ORGANISM] AND (16S[All fields]) AND (nitrogen-fixing[All fields] OR diazotroph[All fields] OR oligonitrotroph[All fields]) OR Frankia[ORGANISM)); for denitrifying bacteria: (bacteria[ORGANISM] AND denitrif[All fields] AND 16S[All fields]), while for nitrifying ones (bacteria[ORGANISM] AND nitrification[All fields] AND 16S[All fields]). Downloaded sequences were converted to BLAST databases with makeblastdb, then the databases were searched with representative sequences from each OTU. BLASTN searches were performed with the following options: -outfmt 6 -max_target_sequences 1 -evalue 0.0001 -perc_identity 97. Alignments with query coverage <99% were filtered out from the resulting tables.

**Methodology of fungal sequences qualification to functional groups.**

Forty top results were inspected, three most relevant were placed in Supplementary Table 2. An organism was regarded as saprophytic when there were no reports on its being an endophyte or a parasite. At least one report of parasitism/pathogenicity was required to qualify an organism to the P group.


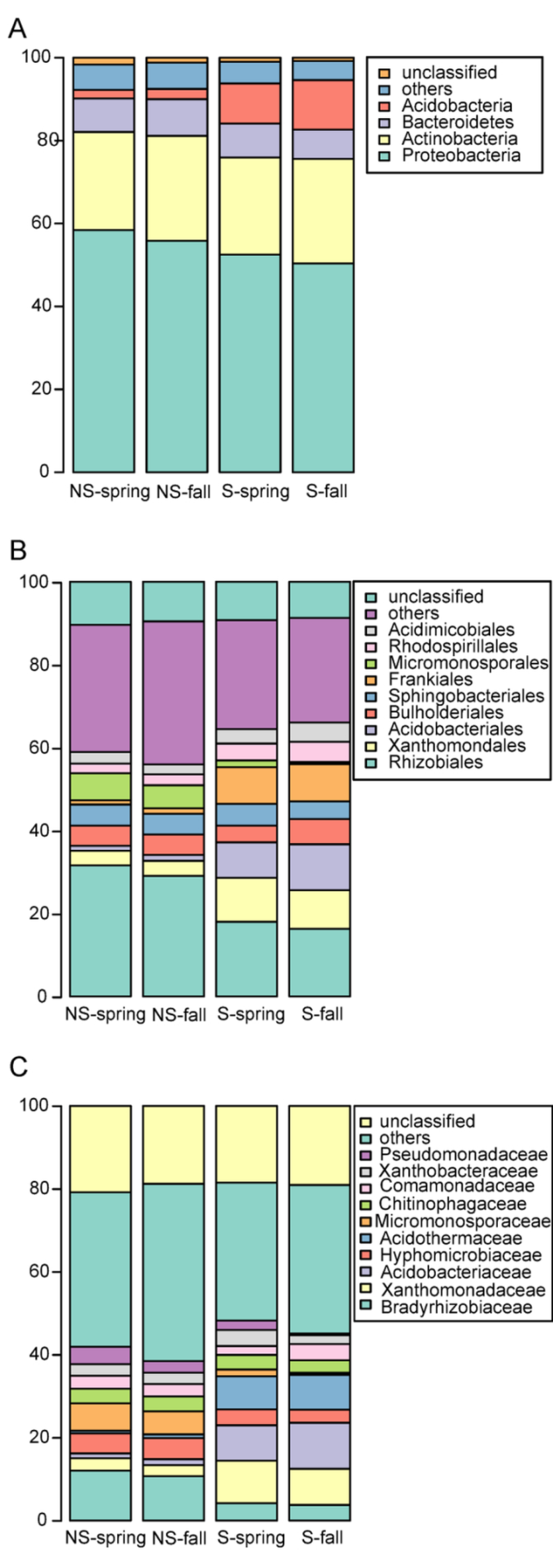


**Supplementary Figure 2.** Bacterial community structure at (**A**) phylum, (**B**) order, (**C**) family level.


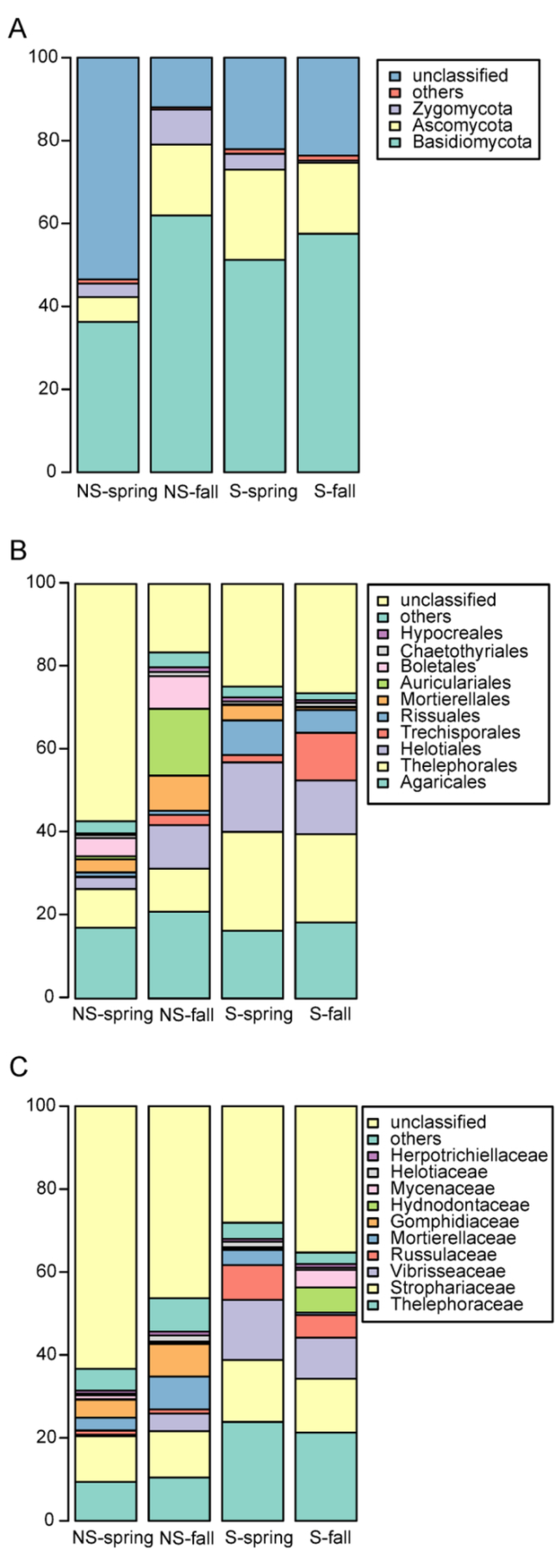


**Supplementary Figure 3.** Fungal community structure at (**A**) phylum, (**B**) order, (**C**) family level.


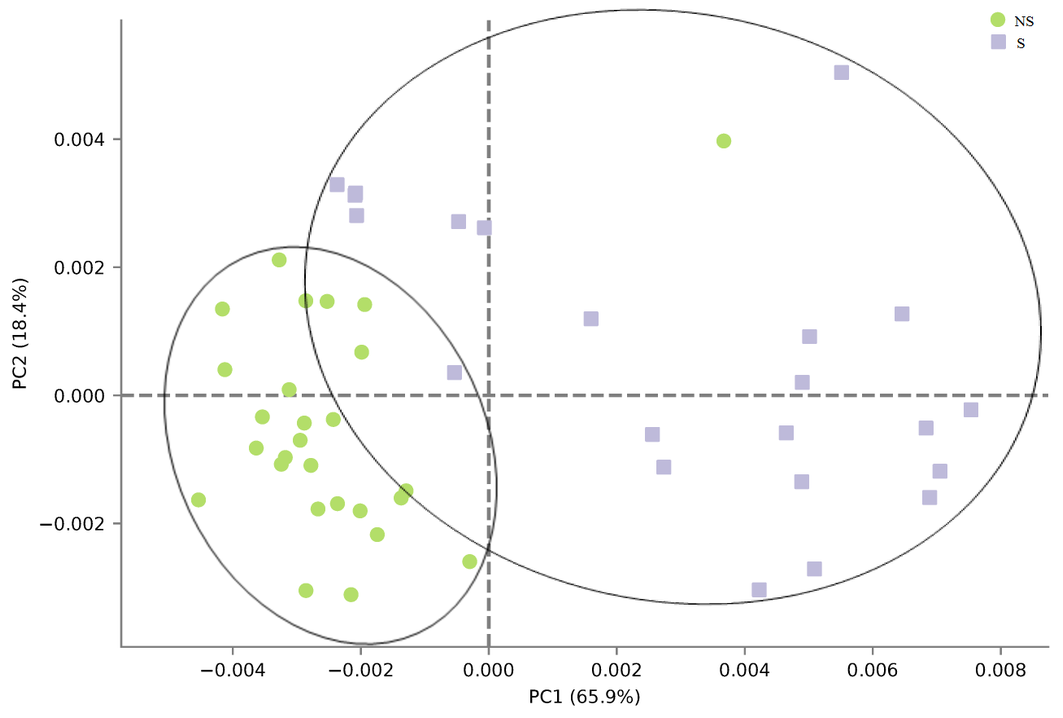
**Supplementary Figure 4.** PCA (Principal Component Analysis) analysis of Bray-Curtis distance matrix based on KO (Kegg Orthology) categories identified by PICRUSt.

**Supplementary Table 1.** Functional diversity measured as the number of KO categories identified by PICRUSt.

| Group | Number of KO categories (mean ± SD) |
| --- | --- |
| NS spring | 5052 ± 226 |
| NS fall | 5093 ± 138 |
| S spring | 5040 ± 193 |
| S fall | 5069 ± 231 |

**Supplementary Table 2.** PICRUSt analysis for bacterial community.

| KO | Function | pathway /role |
| --- | --- | --- |
| K10747 | DNA ligase 1 | DNA replication |
| K03077 | L-ribulose-5-phosphate 4-epimerase | pentose and glucuronate interconversions, ascorbate and aldarate metabolism,  microbial metabolism in diverse environments |
| K09607 | immune inhibitor A | acting on peptide bonds, metalloendopeptidases |
| K10200 | N-acetylglucosamine transport system substrate binding protein | ABC transporters; saccharide, polyol, and lipid transporters |
| K01281 | X-Pro dipeptidyl-peptidase | Acting on peptide bonds (peptidases) |
| K10202 | N-acetylglucosamine transport system permease protein | ABC transporters; saccharide, polyol, and lipid transporters |
| K10201 | N-acetylglucosamine transport system permease protein | ABC transporters; saccharide, polyol, and lipid transporters |
| K09759 | nondiscriminating aspartyl-tRNA synthetase | aminoacyl-tRNA biosynthesis |
| K06880 | erythromycin esterase | acting on ester bonds |
| K14375 | dTDP-L-oleandrosyltransferase | biosynthesis of antibiotics |
| K05551 | 3-oxoacyl-ACP synthase I | tetracycline biosynthesis |
| K05552 | act minimal PKS chain-length factor | tetracycline biosynthesis |
| K00292 | saccharopine dehydrogenase | metabolizm of opinies |
| K04780 | nonribosomal peptide synthetase DhbF | biosynthesis of siderophore group nonribosomal peptides |
| K14368 | L-desosaminyltransferase | biosynthesis of antibiotics |
| K04343 | streptomycin 6-kinase | streptomycin resistance, deactivating enzyme StrAB |
| K01400 | bacillolysin | acting on peptide bonds (peptidases) |
| K01416 | snapalysin | acting on peptide bonds (peptidases) |
| K11949 | 4-(2-carboxyphenyl)-2-oxobut-3-enoate aldolase | polycyclic aromatic hydrocarbon degradation,  microbial metabolism in diverse environments |
| K00272 | D-aspartate oxidase | alanine, aspartate and glutamate metabolism |
| K05550 | benzoate 1,2-dioxygenase beta subunit | benzoate degradation,  fluorobenzoate degradation,  xylene degradation, microbial metabolism in diverse environments, degradation of aromatic compounds |
| K10677 | inulin fructotransferase (DFA-I-forming) | lyases, acting on polysaccharides |
| K10673 | streptomycin 3 kinase | streptomycin resistance |
| K02799 | PTS system, mannitol-specific IIB component | fructose and mannose metabolism |
| K00152 | salicylaldehyde dehydrogenase | naphthalene degradation, microbial metabolism in diverse environments, degradation of aromatic compounds |
| K04117 | cyclohexanecarboxyl-CoA dehydrogenase | benzoate degradation,  microbial metabolism in diverse environments |
| K12675 | clavaminate synthase | clavulanic acid biosynthesis, biosynthesis of antibiotics |
| K08710 | N-isopropylammelide isopropylaminohydrolase | atrazine degradation, microbial metabolism in diverse environments |
| K00999 | CDP-diacylglycerol--inositol 3-phosphatidyltransferase | inositol phosphate metabolism, glycerophospholipid metabolism,  phosphatidylinositol signaling system |
| K12529 | putative selenate reductase FAD-binding subunit | metabolism of other amino acids |
| K14164 | glycyl-tRNA synthetase | aminoacyl-tRNA biosynthesis |
| K07272 | rhamnosyltransferase | glycosyltransferases |
| K12980 | lipid A oxidase | lipopolysaccharide biosynthesis proteins |
| K10236 | trehalose/maltose transport system substrate-binding protein | ABC transporter ; trehalose/maltose transport |
| K10237 | rehalose/maltose transport system permease protein | ABC transporters; rehalose/maltose transport |
| K10239 | trehalose/maltose transport system ATP-binding protein | ABC transporter ; trehalose/maltose transport |
| K11889 | type VI secretion system protein ImpN | transferases |
| K11078 | mannopine transport system permease protein | ABC transporters, Mannopine transport system |
| K00296 | D-nopaline dehydrogenase | arginine and proline metabolism |
| K11077 | mannopine transport system substrate-binding protein | ABC transporters, Mannopine transport system |
| K11080 | mannopine transport system ATP-binding protein | ABC transporters, Mannopine transport system |
| K10856 | acetone carboxylase, gamma subunit | ligases that form carbon-carbon bonds |
| K07291 | CDP-L-myo-inositol myo-inositol phosphotransferase | inositol phosphate metabolism |
| K08651 | thermitase | serine endopeptidases |
| K06027 | vesicle-fusing ATPase | autophagy - yeast,  synaptic vesicle cycle,  GABAergic synapse,  vasopressin-regulated water reabsorption |
| K12554 | alanine adding enzyme | peptidoglycan biosynthesis |
| K00862 | erythritol kinase | transferring phosphorus-containing groups |
| K01318 | glutamyl endopeptidase | quorum sensing |
| K02782 | PTS system, glucitol/sorbitol-specific IIB component | fructose and mannose metabolism, phosphotransferase system (PTS) |
| K01355 | omptin | aspartic endopeptidases |
| K14286 | alanine-glyoxylate aminotransferase 2-ligase | glycerophospholipid metabolism |
| K07824 | benzoate 4-monooxygenase | benzoate degradation,  aminobenzoate degradation |
| K13658 | beta-1,2-mannosyltransferase | hexosyltransferases |
| K07318 | adenine-specific DNA-methyltransferase | site-specific DNA-methyltransferase (adenine-specific) |
| K09722 | 4-phosphopantoatebeta-alanine ligase | beta-alanine metabolism |
| K06982 | pantoate kinase | pantothenate and CoA biosynthesis |
| K00608 | aspartate carbamoyltransferase | alanine, aspartate and glutamate metabolism |
| K07255 | taurine dehydrogenase small subunit | taurine and hypotaurine metabolism |
| K11913 | type VI secretion system protein | biofilm formation |
| K15019 | 3-hydroxypropionyl-coenzyme A dehydratase | energy metabolism |
| K12956 | cation-transporting ATPase V | acting on acid anhydrides -hydrolase |

**Supplementary Table 3.** Functional analysis for identified fungal genera and species.

| **Name of identified species** | **Acc. number in NCBI** | **Source of isolation** | **References in NCBI** | **Group of fungi** | **Potentially**  **salt -tolerant** |
| --- | --- | --- | --- | --- | --- |
| *Tomentella testaceogilva* | KC782512  KC782511  KC782510 | roots of *A. glutinosa* | Nouhra et al., 2014 | M | **-** |
| *Trechisporales* sp. | MF339147  KY228539  KJ786681 | rhizome-associated mycobiome  *Erica* dominans root  EM roots | unpublished | E/M | **-** |
| *Lactarius cyathuliformis* | KX610691  HQ714869  KF133330 | EM fungi  EM fungi  EM fungi | Kałucka et al., 2016  Rochet et al., 2011  Verbeken et al., 2014 | M | **-** |
| *Thelephora alnii* | KM522806 | ectomycorrhizal root tip | unpublished | M | **-** |
| *Trechispora* sp. | MF340207  JQ673207 | rhizome-associated mycobiome  fruit bodies in forest dominated by aspen | unpublished  Breeze et al., 2011 | E/S | **-** |
| *Mycena galopus* | MF926552  KU516421  KY352526 | root *Orthilia secunda*  stem of *Abies alba* seedlings  fine root | Malysheva et al., 2017  unpublished  unpublished | E/S | **-** |
| *Auriculariales* sp. | MF339189  KY228818  AB847046 | rhizome-associated mycobiome  *Erica* dominans root  root of *Enkianthus campanulatus* | unpublished  unpublished  Obase and Matsuda, 2014 | E | **-** |
| *Tomentella ellisii* | DQ974775  HQ406823  AB634269 | roots and sporocarps *Quercus*  ectomycorrhizal root tip *Pinus sylvestris* seedlings  ectomycorrhizal root tips *Pinus massoniana* Lamb. | Smith et al., 2007  Menkis et al., 2012  Huang et al., 2012 | M | **-** |
| *Cadophora orchidicola* | KY271872  KU981161  KR135151 | *Gymnadenia nigra*  *Gymnadenia nigra*  root *Crocus sativus* | Schiebold et al., 2017  unpublished  unpublished | M/E | **-** |
| *Mortierella parvispora* | KU516633  KC009047  KC018414 | root of *Abies alba* seedlings  cave and mine  mycelium | Jankowiak et al., 2016  Zhang et al., 2014  Wagner et al., 2013 | E/S | **-** |
| *Cladophialophora chaetospira* | MF339442  KY680415  KF359558 | rhizome-associated mycobiome  decaying shell of babassu coconut (*Orbignya phalerata*)  root of Eastern Hemlock | unpublished  Nascimento et al., 2017  Baird et al., 2014 | E | **-** |
| *Lactarius obscuratus* | KY659397  HQ714870  GU234159 | roots of *Eucalyptus globulus*  roots of *A. glutinosa*  sporocarp in Svalbard | unpublished  Rochet et al., 2011  Geml et al., 2012 | M | **-** |
| *Varicosporium* sp. | KX100406  KJ542329 | arctic driftwood  root of *Rhodiola crenulata* | Blanchette et al., 2016  Cui et al., 2015 | E | **-** |
| *Acephala applanata* | HM347318  KU516423  DQ274578 | *Pinus sylvestris*  root of *Abies alba* seedlings  *Picea abies* | Zaffarano et al., 2010  Jankowiak et al., 2016  Gruning et al., 2017 | E | **-** |
| *Meliniomyces vraolstadiae* | HQ157928  AJ292200  FN678887  HQ157883 | *Picea glaca*  ectomycoorhizae of *Betula pubescens*  ectomycorrhizal root tip  *Pinus sylvestris*  *Abies balsamifera* | Kernaghan and Patriquin 2011  Vrasland et al., 2000  unpublished  Kernaghan and Patriquin 2011 | M/E | **-** |
| *Exophiala equine* | JF747099 | water | de Hoog et al., 2011 | P | **-** |
| *Delicatula integrella* | KR673538  AM946446 | fruit bodies  fruit bodies | Kim et al., 2015  Saar et al., 2009 | S | **-** |
| *Mortierella elongate* | JX155654  KY322664  KP017884 | soil  *Pinus heldreichii* fine roots  endophyte of halophytes | Lazarevic and Menkis, 2017  Khalmuratova et al., 2015  unpublished | E | **+** |
| *Mortierella clonocystis* | JX975929  HQ667479  NG042554 | mycelium  mycelium  mycelium | Wagner et al., 2013  Petkovits et al., 2011  Petkovits et al., 2011 | S | **-** |
| *Mortierella gamsii* | KY305027  KC008853  DQ093723 | thawed soil from a pine forest  cave and mine  *Pinus sylvestris* decayed root | Micalizzi et al., 2011  Zhang et al., 2014  Menkis et al., 2006 | S | **-** |
| *Cistella* sp. | KY322651  KT270225  GU395497 | *Pinus heldreichii* fine roots  surface-sterilized, asymptomatic roots  *Saussurea involucrata* Kar. et Kir. | Lazarevic and Menkis, 2017  Glynou et al., 2015  unpublished | E | **-** |
| *Mortierella globulifera* | JX976165  JQ014040  HQ667455 | mycelium  mycelium  mycelium | Wagner et al., 2006  Schoch et al., 2012  Petkovits et. al., 2011 | S | **-** |
| *Epicoccum nigrum* | AY521446  HQ846571  KZ107868 | *Pinus*  *Panicum virgatum*  soil | Wang et al., 2004  Kleczewski et al., 2012  Fokin et al., 2017 | E | **-** |
| *Mycena stylobates* | JF908439 | mycelium from macrofungal herbarium | - | U | **-** |
| *Papiliotrema flavescens* | MG367282  LT627406  KY940306 | *Castanopsis xerox*  flower  flower of *Justicia adhatoda* L. | unpublished  Ribeiro et al., 2017  unpublished | E | **-** |
| *Thanatephorus* sp. | MF337379  KT269974  KU847462 | rhizome-associated mycobiome  surface-sterilized, asymptomatic roots of *Microthlaspi perfoliatum*  root of orichid *Oeceoclades maculata* | unpublished  Glynou et al., 2015  unpublished | E | **-** |
| *Pezicula radicicola* | KY381837  KR859029 | stem of *Sophora* *tonkinensis Gagnep*.  *Quercus robur* | unpublished  Chen et al., 2016 | E | **-** |
| *Cryptococcus tephrensis* | AF317208    KX096667  JN400782 | soil from a salt farm on the Taean peninsula in Korea  endophyte on plant, phylosphere  glacier surface snow | Vishniac, 2002  unpublished  unpublished | S/E | **+** |
| *Solicoccozyma terricola* | EU289366  MF927659  KX773541 | soil, plant, mycelium  soil  soil from Antarctica | unpublished  unpublished  Martorell et al., 2017 | S/E | **-** |
| *Talaromyces albobiverticillius* | KP017805  KF114737 | root of halophytes  mycelium from BS-KNAW Fungal Biodiversity Centre culture collection | Khalmuratova et al., 2015  Frisvad et al., 2013 | E | **+** |
| *Cladophialophora* sp. | MF588932  KY315565  MF339988 | *Panax notoginseng*  coconut palms  *Panax notoginseng* | unpublished  unpublished  unpublished | E | **-** |
| *Archaeorhizomyces* sp. | MF342374  KX463656  JF836024 | rhizome-associated mycobiome  *Glycyrrhiza glabra*  soil | unpublished  unpublished  Rosling et al., 2011 | S/E | **-** |
| *Remispora stellata* | KM272364  KM272365  HQ111017 | driftwood on Arctic Ocean  marine water  unknown | Rama et al., 2014  Rama et al., 2014  Sakayaroj et al., 2011 | S | **+** |
| *Mucor hiemalis* | KY419554  KY034430  KX620496 | *Glycyrrhiza gabra*  olive fruit  seawater | unpublished  unpublished  unpublished | E | **+** |
| *Phialocephala fortinii* | DQ335464  DQ417832   DQ417861 | *Pinus sylvestris*  *Picea abies*  *Picea abies* | Gruning et al., 2008  Gruning et al., 2008  Gruning et al., 2008 | E | **-** |
| *Brevicellicium exile* | KP814539  HE963778 | on litter or well decayed wood  in basidiome on host pinaceous forest  *Buxus sempervirens* | unpublished  Telleria et al., 2013 | S | **-** |
| *Helotiales* sp. | KY271866  MG195551  KX389112 | *Liparis loeselii*  *Nothofagus* sp.  *Ozark chinquapin* | Schiebold et al., 2017  unpublished  unpublished | E | **-** |
| *Cryptococcus podzolicus* | LC155938  HG737350  KU702589 | soil  soil  forest soil | unpublished  Schulze et al.,2014  unpublished | S | **-** |
| Archaeorhizomycetes sp. | MF330167  KC876138  M061054 | rhizome-associated mycobiome  *Betula papyrifera*  *Allanblackia stuhlmannii* | unpublished  Kernaghan and Patriquin, 2015  unpublished | E | **-** |
| *Cortinarius alnetorum* | FJ039534  JQ749628  FM993281 | speicment  *Alnus* sp.  *Alnus* sp. | Harrower et al., 2011  unpublished  unpublished | M | **-** |
| *Ilyonectria robusta* | JF735722  KF157969  KM015299 | *Thymus* sp.  grapevine  root of Asian Ginseng | Cabral et al., 2012  dos Santos et al., 2014  Lu et al., 2015 | P | **-** |
| *Mycena angusta* | JF908371 | unknown | Osmundson et al., 2013 | U | **-** |
| *Leotiomycetes* sp. | KY591642  KU597359  KX602710 | rhizome-associated mycobiome  root *Panicum virgatum*  bark tissue *Thuja occidentalis* | unpublished  unpublished  unpublished | E | **-** |
| *Pyrenochaetopsis leptospora* | KX712403  NR119958  JF740262 | *Pyrenochaetopsis leptospora*  *Secale cereale*  *Pyrenochaetopsis leptospora* | Soler-Hurtado et al., 2016  de Gruyter et al., 2013  de Gruyter et al., 2013 | P | **-** |
| *Lachnum virgineum* | KY322550  MF339307  JQ272454 | *Pinus heldreichii* fine root  rhizome-associated mycobiome  Rhododendron | Lazarevic and Menkis 2017  unpublished  Baird et al., 2014 | E | **-** |
| *Capronia* sp. | MF340073  AY953957  KP035001 | rhizome-associated mycobiome  unknown  tap water | unpublished  unpublished  unpublished | U | **-** |
| *Exophiala opportunistica* | KP347969  JN112409 | rock  water | unpublished  [de](https://www.ncbi.nlm.nih.gov/pubmed/?term=de%20Hoog%20GS%5BAuthor%5D&cauthor=true&cauthor_uid=22403476) Hoog et al., 2011 | P | **-** |
| *Alnicola* sp. | MG255256  HQ714802 | soil from mycorrhizosphere  *Alnus alnobetula* subsp. *alnobetula* | unpublished  Moreau et al., 2011 | M | **-** |
| *Phomopsis columnaris* | KY318496  KX610404  KU204666 | *Sceletuim* leaves, root  root of *Fragaria vesca*  *Cupania livida* | unpublished  unpublished  unpublished | E | **-** |
| *Cortinarius bibulus* | EU821677  FM993285 | fruit body  *Alnus* sp. | unpublished  Harrower et al., 2011 | M | **-** |
| *Cyphellophora* sp. | KY322530  KY589799  KT270132 | *Pinus heldreichii* fine roots  rhizome-associated mycobiome  surface-sterilized, asymptomatic roots | Lazarevic and Menkis, 2017  unpublished  Glynou et al., 2015 | E | **-** |
| *Microscypha* sp. | MG434781  MF336663 | root tips  rhizome-associated mycobiome | unpublished  unpublished | E | **-** |
| *Mortierella sclerotiella* | KU320631  JQ346223 | soil from Orchard  *Myrceugenia ovata* var. *Nanophylla* | unpublished  Vaz et al., 2014 | S | **-** |
| *Penicillium canescens* | GQ996946  KJ948114  FJ860892 | mycelium  mycelium  mycelium | unpublished  Volkov et al., 2014  unpublished | S | **-** |
| Glomeromycetes sp. | KF359634  JQ272369  AB824133 | root  Rhododendron  volcanic ash soil | Baird et al., 2014  Baird et al., 2014  unpublished | M | **-** |
| *Calyptella capula* | - | - | - | U | **-** |
| *Metarhizium carneum* | KY320621  KU555979  KR296911 | soil  root of coastal plant  soil | unpublished  unpublished  Ottenheim et al., 2015 | S/E | **-** |
| *Minimelanolocus obscurus* | KR215616  KR215611  KR215606 | decaying wood submerged in a stream  decaying wood submerged in a stream  decaying wood submerged in a stream | Liu et al., 2015  Liu et al., 2015  Liu et al., 2015 | U | **-** |
| *Hyalodendriella betulae* | EU040232  FJ755263 | *Alnus glutinosa*  unknown | Crous et al., 2007  unpublished | P | **-** |
| *Trechispora stevensonii* | UDB016467 | unknown | unpublished | U | **-** |
| *Oliveonia pauxilla* | HQ441577  HQ441576 | unknown  unknown | Roberts, 1999  Roberts, 1999 | U | **-** |
| *Dactylella mammillata* | KT215290  DQ093778 | wood of *Fagus sylvatica*  *Pinus sylvestris* decayed root | Menkis et al., 2006  Menkis et al., 2006 | E | **-** |
| *Tetracladium maxilliforme* | KX610446  HM036615  KC989085 | root of *Fragaria vesca*  root tip of *Pinus sylvestris*  root of *Triticum aestivum* | unpublished  Menkis and Vasaitis, 2011  unpublished | E | **-** |
| *Boeremia exigua* | LT838277  KX284747  KR653206 | cotton leaves  *Morus alba* L.  Ipomoea batatas | unpublished  unpublished  unpublished | P | **-** |
| *Thelephora* sp. | MF352784  KY090906  KR819122 | ectomycorrhiza of *Pinus sylvestris*  fruiting body  ectomycorrhizal root of *Gilbertiodendron dewevrei* | Suz et al., 2017  unpublished  unpublished | M | **-** |
| *Phialocephala* sp. | MG434779  MF948655  KU986817 | root tips  roots of *Pinus monticola*  roots of *Vaccinium myrtillus* L. | unpublished  unpublished  Hamim et al., 2017 | E | **-** |
| *Hypomyces rosellus* | AF160242 | species growing on wood-decaying | Pỡldmaa et al., 2000 | S | **-** |
| *Naucoria alnetorum* | - | - | - | U | **-** |
| *Trechispora invisitata* | KP814425 | on litter or well decayed wood in pinaceous forest | unpublished | S | **-** |
| *Operculomyces laminatus* | - | - | - | U | **-** |
| *Piskurozyma filicata* | - | - | - | U | **-** |
| *Naucoria subconspersa* | HQ714741  JN943973 | *A. glutinosa*  unknown | Rochet et al., 2011  Schoch et al., 2012 | M | **-** |
| *Crypococcus victoriae* | KT875333  KC433849  LC125573 | soil  sediment  arctic soil | unpublished  Turchetti et al., 2013  unpublished | S | **-** |
| *Metapochonia* sp. | - | - | - | U | **-** |
| *Inocybe rufoalba* | KX602273  JF908207 | dried basidioma  Venice Museum Fungal Collection | Kalucka et al., 2016  Osmundson et al., 2013 | M | **-** |
| *Atheliales* sp. | MF337411  KY462660  KY462544 | rhizome-associated mycobiome  sporocarp  sporocarp | unpublished  unpublished  unpublished | S | **-** |
| *Neonectria* sp. | KY318492  KX610379  KT270041 | sceletuim leaves, roots  roots of *Fragaria vesca*  surface-sterilized, asymptomatic roots of *Microthlaspi perfoliatum* | unpublished  unpublished  Glynou et al., 2015 | E | **-** |
| *Epithele typhae* | - | - | - | U | **-** |
| *Pseudogymnoascus roseus* | HM036614  JX077081  KJ755524 | root tip of *P. sylvestris*  wetland sediment  soil | Menkis and Vasaitis, 2011  Wu et al., 2013  unpublished | E/S | **-** |
| *Amanita muscaria* | DQ822791  DQ179118  KU693323 | mushroom  ectomycorrhizal fruitbody  roots of *Acacia melanoxylon* | Peay et al., 2007  unpublished  unpublished | M | **-** |
| *Sarocladium bactrocephalum* | HG965007  KC798431  KM096139 | *Homo sapiens*  banana nectar  seawater | Giraldo et al., 2015  unpublished  Punzer et al., 2015 | S | **+** |
| *Eucasphaeria sp.* | KX774173  HQ914909 | leaf of *Platycladus orientalis*  marine macroalgae | unpublished  unpublished | E | **+** |
| *Scopuloide hydnoides* | LN611029  EU118665  AJ406574 | genomic DNA  genomic DNA  genomic DNA | Kuuskeri et al., 2015  Larson, 2007  unpublished | S | **-** |
| *Alnicola inculta* | AY900099  AY900098  AY900097 | ectomycorrhizal mushroom  ectomycorrhizal mushroom  ectomycorrhizal mushroom | Moreau et al., 2006  Moreau et al., 2006  Moreau et al., 2006 | M | **-** |
| *Phaeomyces* sp. | KU934003 | surface-sterilized, asymptomatic roots  *Salicornia emerici* | unpublished | E | **+** |
| *Typhula* sp. | KY235233  KY353750  LC005087 | *Acer platanoides* leaves  soil from restored and/or remnan tallgrass prairie  on dead rotting leaves o Stilbocarpa | unpublished  unpublished  unpublished | S | **-** |
| *Oidiodendron* sp. | KY591921  HM208721  FJ911880 | rhizome-associated mycobiome  *Rhododendro*n sp.  marine macroalgae from Antarctica | unpublished  Loque et al., 2010  unpublished | E | **+** |
| *Ophiosphaerella* sp. | MF341231  KY987527  KT270300 | rhizome-associated mycobiome  dark septate endophyte (DSE)  surface-sterilized, asymptomatic roots of *Microthlaspi perfoliatum* | unpublished  unpublished  Glynou et al., 2016 | E | **-** |
| *Chrysosporium pseudomerdarium* | KC864749  NR145209  AB361652 | marine sediment  beach sand  beach sand | unpublished | S | **-** |
| *Clavaria falcata* | JQ415961  JQ415945  GU234148 | pasture  soil under young *Fagus*  arctic soil and sporocarps | unpublished  unpublished  Geml et al., 2012 | S | **-** |
| *Mortierella exigua* | KP744419  JX045738  HQ630329  FJ161943 | soil  rhizosphere of *Fragaria virginiana*  genomic DNA  soil | unpublished  unpublished  Nagy et al., 2011  unpublished | S | **-** |
| *Imleria badia* | KY693727  LN877746  KF007935 | roots of *Pinus pinaster*  sporocarp of *Picea abies*  fruiting body | unpublished  Cejpkova et al., 2016  Baird et al., 2014 | M | **-** |
| *Oidiodendron maius* | EU386164  KJ158156  JF304942 | genomic DNA  genomic DNA  ericoid mycorrhizal hair root of *Rhododendron* sp. | Martino et al., 2002  unpublished  Vohnik et al., 2015 | M | **-** |
| *Protoventuria* sp. | AB831875 | detected from the root of Neottia (Orchidaceae) species | unpublished | E | **-** |
| *Trichocladium opacum* | KU516597  KF850373  FJ903366 | root of *Abies alba*  roots from pine wilt disease areas  decayed wood | Jankowiak et al., 2016  unpublished  Arhipova et al., 2011 | P | **-** |
| *Tausonia pullulans* | KX620505  KC433855  KY033106 | seawater  supraglacial sediments  soil | unpublished  Turchetti et al., 2013  Martorell et al., 2017 | S | **+** |
| *Mycoarthris* sp. | KT270092  J X982481  KJ735002 | surface-sterilized, asymptomatic roots of *Microthlaspi perfoliatum*  endophyte of *Arabidopsis thaliana*  plateau grassland soil | Glynou et al., 2015  Garcia et al., 2013  unpublished | E | **-** |
| *Lasiosphaeria lanuginosa* | KX394786  KJ407065 | *endolichenic fungus associated with genus Cladonia*  needles of *Pinus radiata* | unpublished  unpublished | E | **-** |
| *Cotylidia* sp. | - | - | - | U | **-** |

**Functional groups**

M - mycorrhizal fungus

E - endophyte

S - saprobe

P - pathogen

U - unknown, lack of sufficient data in NCBI to assign

**Potentially salt-tolerant**

Fungus found in the seawater, saline soil or as endophyte of halophytes (+)

There is no information about founding in the seawater, saline soil or as endophyte of halophytes (-)
